# Plastoquinone synthesis inhibition by tetrabromo biphenyldiol as a widespread algicidal mechanism of marine bacteria

**DOI:** 10.1038/s41396-023-01510-0

**Published:** 2023-09-08

**Authors:** Zenghu Zhang, Dehai Li, Ruize Xie, Ruoyu Guo, Shailesh Nair, Huan Han, Guojian Zhang, Qun Zhao, Lihua Zhang, Nianzhi Jiao, Yongyu Zhang

**Affiliations:** 1 Key Laboratory of Biofuels, Shandong Provincial Key Laboratory of Energy Genetics, Qingdao Institute of Bioenergy and Bioprocess Technology, Chinese Academy of Sciences, Qingdao, 266101 China; 2 Shandong Energy Institute, Qingdao, 266101 China; 3 Qingdao New Energy Shandong Laboratory, Qingdao, 266101 China; 4 University of Chinese Academy of Sciences, Beijing, 100049 China; 5 School of Medicine and Pharmacy, Ocean University of China, Qingdao, 266003 China; 6 CAS Key Laboratory of Separation Science for Analytical Chemistry, National Chromatographic R. &A. Center, Dalian Institute of Chemical Physics, Chinese Academy of Sciences, Dalian, 116023 China; 7 State Key Laboratory of Marine Environmental Science, Xiamen University, Xiamen, 361101 China

**Keywords:** Microbial biooceanography, Microbial biooceanography

## Abstract

Algae and bacteria have complex and intimate interactions in the ocean. Besides mutualism, bacteria have evolved a variety of molecular-based anti-algal strategies. However, limited by the unknown mechanism of synthesis and action of these molecules, these strategies and their global prevalence remain unknown. Here we identify a novel strategy through which a marine representative of the *Gammaproteobacteria* produced 3,3’,5,5’-tetrabromo-2,2’-biphenyldiol (4-BP), that kills or inhibits diverse phytoplankton by inhibiting plastoquinone synthesis and its effect cascades to many other key metabolic processes of the algae. Through comparative genomic analysis between the 4-BP-producing bacterium and its algicidally inactive mutant, combined with gene function verification, we identified the gene cluster responsible for 4-BP synthesis, which contains genes encoding chorismate lyase, flavin-dependent halogenase and cytochrome P450. We demonstrated that in near in situ simulated algal blooming seawater, even low concentrations of 4-BP can cause changes in overall phytoplankton community structure with a decline in dinoflagellates and diatoms. Further analyses of the gene sequences from the Tara Oceans expeditions and 2750 whole genome sequences confirmed the ubiquitous presence of 4-BP synthetic genes in diverse bacterial members in the global ocean, suggesting that it is a bacterial tool potentially widely used in global oceans to mediate bacteria-algae antagonistic relationships.

## Introduction

Phytoplankton (microalgae), the most important primary producers in the ocean [1], are inseparable from bacteria, and their long-term coexistence at the evolutionary scales has shaped their complex relationships ranging from mutualism, antagonism, and competition to parasitism [2]. Such interactions are also known to play important roles in regulating the biogeochemical cycles in the ocean [3, 4].

Aside from the inherent tendency for mutualism during the long-term coexistence of algae and bacteria [5], antagonistic relationships between them also frequently occur under certain environmental conditions, particularly in eutrophic environments or during the late stage of algal blooms [6]. For example, a high proportion of algae-killing (or algicidal) bacteria often emerge in the late stage of an algal bloom and these are thought to be involved in the cessation of the bloom [4]; Even in a laboratory-based algae and bacteria co-culture system, some of the bacteria inhibited or killed the algae, even though the whole bacterial community did not significantly inhibit algal growth [6]. This suggests that the antagonistic relationship between algae and bacteria might be latent, but it can be stimulated under specific conditions. The antagonistic interaction between bacteria and algae in the ocean is critical, not only for the acquisition of resources, but also for the overall stability of the ocean ecosystem.

Algicidal bacteria are of potential importance from an applied perspective because of their potential for preventing algal blooms [7, 8]. Such bacteria can disrupt the cell wall, or inhibit the cell division or motility of algae [4]. However, the most common mode of action of algicidal bacteria is to secrete small molecular substances, such as *p*-coumaric acid, alkaloids, polyketides, terpenes, and fatty acids [4]. A complex interaction between algae and bacteria is based on the exchange of thousands of molecules, where the accumulation of certain molecules can control or transform their interactions [9, 10]; however, to date very few of these molecules have been characterized. Therefore, the identification and characterization of these active molecules, and determining how they function, are important for improving our understanding of the complex phytoplankton-bacteria interactions in the ocean.

Here we report the discovery of a marine member of the *Gammaproteobacteria* with broad-spectrum algicidal activity against phytoplankton. The algicidal effect is due to a previously unknown extracellularly secreted molecule, identified as 3,3’,5,5’-tetrabromo-2,2-biphenyldiol (4-BP). The gene cluster for 4-BP biosynthesis and its algicidal mechanisms, as well as the varied responses of different phytoplankton taxa in natural seawater when exposed to 4-BP, were also examined. Moreover, the algicidal mechanism of 4-BP and the global distribution of the 4-BP biosynthesis gene cluster in oceanic microbiomes were explored to elucidate their prevalence against phytoplankton. Together, our findings reveal a potentially widely used bacteria-made molecule against phytoplankton in the ocean.

## Results

### Microbulbifer sp. RZ01 inhibited a variety of phytoplankton by the extracellular secretion of 3,3’,5,5’-tetrabromo-2,2’-biphenyldiol (4-BP)

**Fig. 1:**
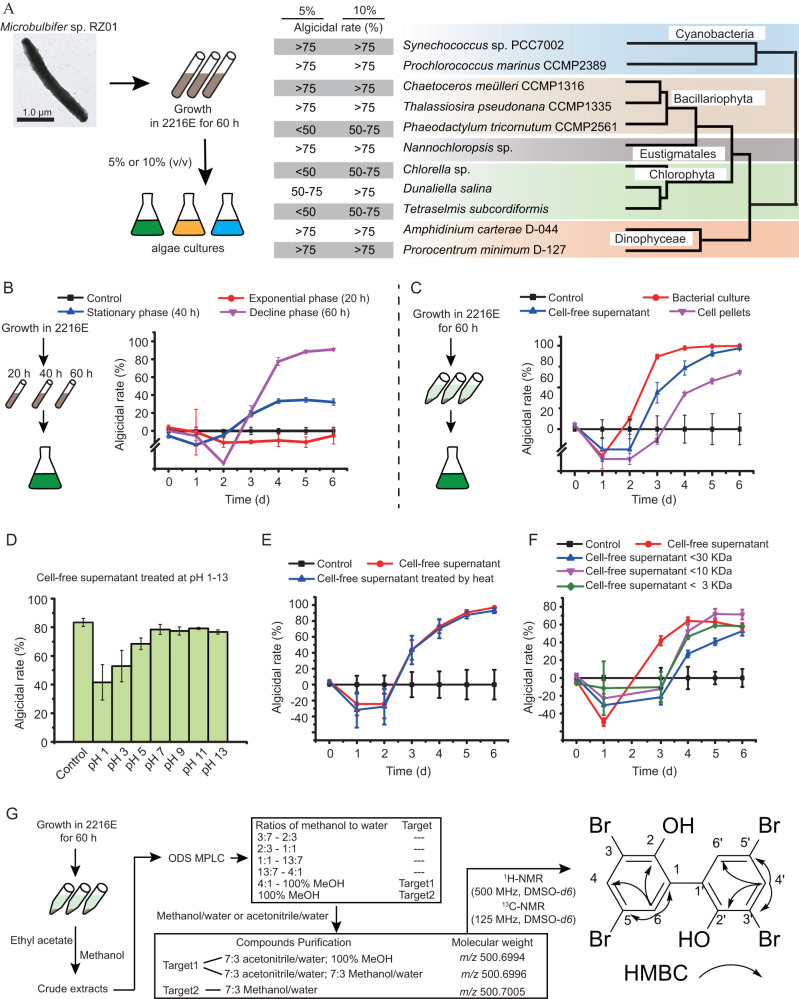
The characteristics of the algicidal active substance in *Microbulbifer* sp. RZ01, and its isolation and identification. **A** The algicidal activity of RZ01 against different algal species. **B** The algicidal effect of RZ01 on differential growth phases of *Synechococcus* sp. PCC7002. **C** The mode of algicidal effect of the RZ01 on *Synechococcus* sp. PCC7002. **D** The algicidal effect of the bacterial supernatant at different pH on *Synechococcus* sp. PCC7002. **E** The algicidal effect of heat-treated supernatant on *Synechococcus* sp. PCC7002. **F** The algicidal effect of the supernatant after centrifugation (with differential size ultrafiltration tubes) on *Synechococcus* sp. PCC7002. **G** Schematic to show the isolation and identification procedures used for this algicidal compound.

*Microbulbifer* sp. RZ01 showed broad algicidal activity against all 11 of the cultivated algal strains affiliated with *Bacillariophyta*, *Chlorophyta*, *Dinoflagellata*, *Cyanophyta*, and *Ochrophyta* that we tested (Fig. 1A, Table S1). The cell-free supernatant of the strain RZ01 culture showed a higher algicidal effect than the cell pellet, and the algicidal effect of the supernatant was more pronounced in the stationary and decline phases than in the exponential phase. These results indicate that strain RZ01 can lyse algae more efficiently in the late growth phase via the extracellular secretion of an active substance (Fig. 1B, C). This active substance had strong structural stability and remained active even at high temperature (i.e., boiling at 100 °C) and extreme ranges of pH (i.e., pH 1–13). In addition, we showed that the molecular weight of the substance is <3 kDa (Fig. 1D–F).

We further isolated the active substance from the supernatant using different fractionation processes, for example, medium-pressure preparation liquid chromatography (MPLC) and high-performance liquid chromatography (HPLC), and initially found three potentially active substances. Subsequent nuclear magnetic resonance (NMR) and mass spectrometry analysis revealed that they all had almost the same molecular weight (i.e., 501.7005, 501.6996, and 501.6994), and heteronuclear multiple bond correlation (HMBC) spectroscopy revealed that they had same correlations between the hydrogen (^1^H) and carbon (^13^C) signals, indicating that they were the same compound. Ultimately, the compound was identified as C_12_H_6_O_2_Br_4_ with a symmetrical structure, i.e., 3,3’,5,5’-tetrabromo-2,2’-biphenyldiol (4-BP) (Fig. 1G).

### Three genes in a gene cluster are responsible for the synthesis of 4-BP

**Fig. 2:**
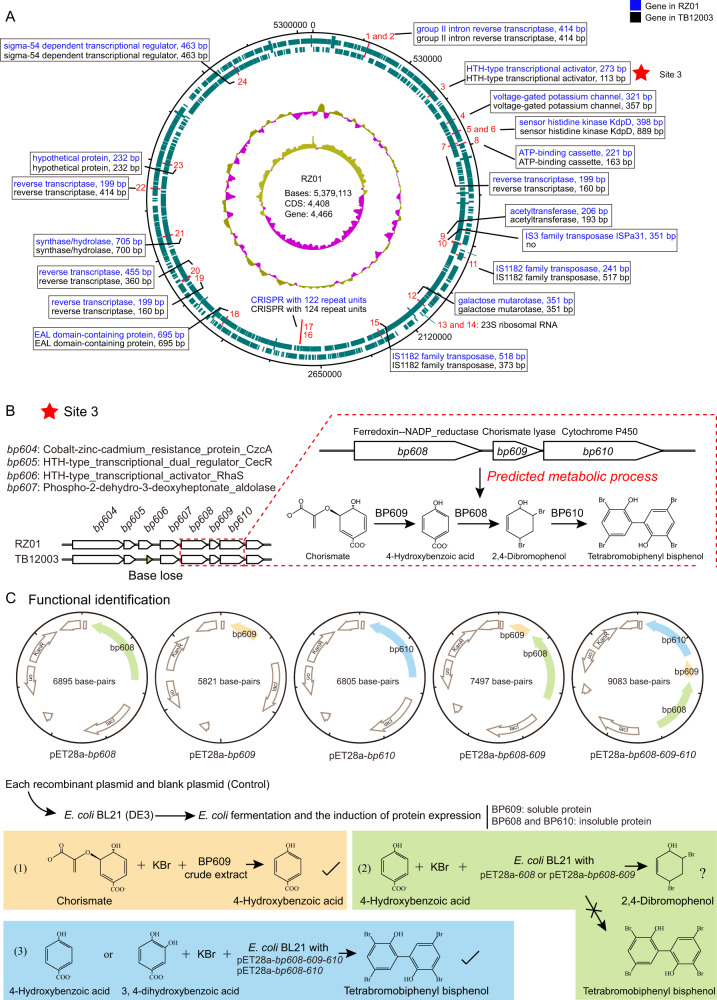
Identification and functional verification of key genes in the synthesis of 4-BP in *Microbulbifer* sp. RZ01. **A** Comparing the genomes of *Microbulbifer* sp. RZ01 and TB12003 and their differential loci. From the outside-in: the first and second circles indicate predicted coding regions on the plus and minus strands, respectively; the third and fourth circles represent percent G + C content and GC skew plot, respectively. The distinct sites between RZ01 and TB12003 are marked labeled with numbers 1–24. **B** The predicted gene cluster, *bp608*-*610*, responsible for 4-BP synthesis in *Microbulbifer* sp. RZ01. A base loss at site 3 resulted in the absence of an HTH-type transcriptional activator in TB12003. Site 3 was located upstream of a potential gene cluster including four genes identified as encoding phospho-2-dehydro-3-deoxyheptonate aldolase (BP607), ferredoxin-NADP reductase (BP608), 4-hydroxybenzoate synthetase (BP609), and cytochrome P450 (BP610). The *bp608*, *bp609* and *bp610* genes were predicted to be involved in the conversion of chorismate to 4-BP, based on previous research on the biosynthesis of polybrominated aromatic organic compounds (Agarwal et al. 2014). **C** Heterologous expression and functional validation of the *bp608*, *bp609* and *bp610* genes, which are responsible for the synthesis of 4-BP. Protein BP609 could convert the chorismate to 4-hydroxybenzoic acid in the presence of bromide ions. The function of BP608 for the conversion of 4-hydroxybenzoic acid to 2, 4-dibromophenol was not determined, however, the co-expression of BP608 and BP610 could convert 4-hydroxybenzoic acid or 3, 4-dihydroxybenzoic acid to 4-BP in the presence of bromide ions.

In addition to *Microbulbifer* sp. RZ01, we also made use of *Microbulbifer* sp. TB12003, which has the same 16 S rRNA gene sequence, but without any algicidal activity (Supplementary Fig. S1). Complete genome sequencing revealed that RZ01 and TB12003 share 99.95% genomic similarity (average nucleotide identity). Thus, we temporarily regarded RZ01 as a wild algicidal strain, and TB12003 as a natural mutant strain that had lost its inhibitory activity against algae. A comparison of the genomes of these two *Microbulbifer* strains (i.e., RZ01 - algicidal and TB12003 - non-algicidal) revealed the key genes that are associated with 4-BP synthesis. We found that RZ01 and TB12003 possess 4466 and 4461 genes, respectively, with 24 distinct sites (Fig. 2A). With the exception of site 3, which was located upstream of a gene cluster, most of these distinct sites were associated with reverse transcriptases, transposases, and CRISPR, or they did not affect protein function (Fig. 2A, B). In the genome of strain TB12003 (unlike in strain RZ01), a base loss at site 3 resulted in the absence of an HTH-type transcriptional activator. Its downstream gene cluster included four genes identified as encoding phospho-2-dehydro-3-deoxyheptonate aldolase (BP607), ferredoxin-NADP reductase (BP608), 4-hydroxybenzoate synthetase (BP609), and cytochrome P450 (BP610) (Fig. 2B). Furthermore, the *bp608, bp609* and *bp610* genes were predicted to be involved in the conversion of chorismate to 4-BP, based on previous research on the biosynthesis of polybrominated aromatic organic compounds [11]. The conversion of chorismate to 4-BP involves three processes, as follows: conversion of chorismate to 4-hydroxybenzoic acid (catalyzed by 4-hydroxybenzoate synthetase), conversion of 4-hydroxybenzoic acid to 2,4-dibromophenol (by ferredoxin-NADP reductase) and conversion of 2,4-dibromophenol to 4-BP (by cytochrome P450).

Heterologous expression experiments confirmed that protein BP609 could convert the chorismate to 4-hydroxybenzoic acid in the presence of bromide ions (Fig. 2C). As the BP608 and BP610 proteins are insoluble in water, we added 4-hydroxybenzoic acid and bromide ions to *Escherichia coli* cultures with the pET28a-*bp608* or pET28a-*bp608*-*bp609* vectors, but the production of 2,4-dibromophenol was not detected, which may be due to the following reasons: (1) some reports revealed that 2,4-dibromophenol has an inhibitory effect on *E. coli* [12, 13], which may limit the accumulation of 2,4-dibromophenol in *E. coli* cultures; (2) the use of recombinant *E. coli* cultures complicates the reaction system and makes detection of the substance difficult compared to the use of pure protein. The difficulty in obtaining soluble BP608 was similar to that in the study of halogenases homologous to BP608 [14]; (3) this halogenase BP608 might not only convert 4-hydroxybenzoic acid to 2,4- dibromophenol, but also 2,4,6-tribromophenol, which may reduce the amount of 2,4-dibromophenol in the system [11]. In any case, Moore et al. succeeded in obtaining protein Bmp5 homologous to BP608 and demonstrated that such enzymes could convert 4-hydroxybenzoic acid to 2,4-dibromophenol, providing indirect evidence for the putative function of BP608 [11]. However, the co-expression of BP608, BP609 and BP610 could convert 4-hydroxybenzoic acid to 4-BP in the presence of bromide ions. This indicates that the simultaneous expression of the *bp608*, *bp609* and *bp610* genes could synthesize 4-BP in the presence of chorismate and bromide ions (Fig. 2C). TB12003 also contained these three genes but did not show any algicidal activity. However, considering that a gene (*bp606*, which encodes a transcriptional activator) is missing at site 3 in this mutant strain, we suggest that this gene may be associated with 4-BP synthesis, but the specific mechanism is unclear.

### 4-BP synthesis genes are widely distributed in global oceanic microorganisms

**Fig. 3:**
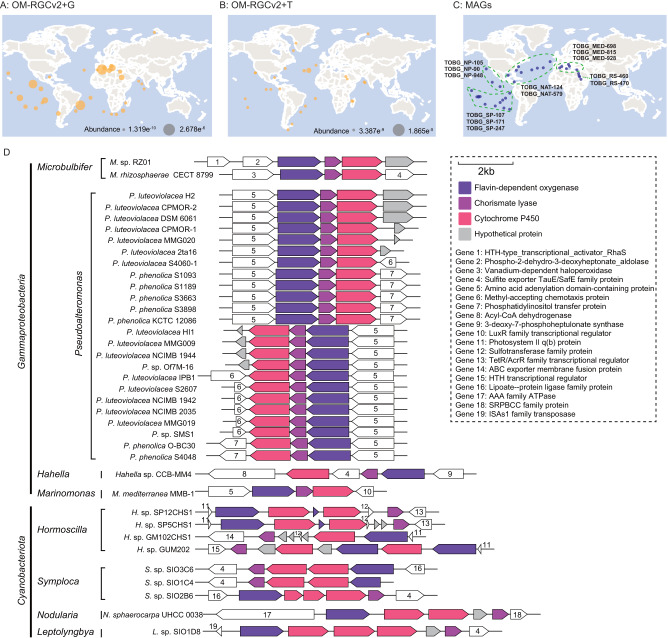
Distribution of the 4-BP synthesis genes in the global oceanic microorganisms. **A** The global marine distribution of 4-BP synthetic genes based on OM-RGCv2_metaG dataset analysis. The dots represent the stations where the three genes are present simultaneously and the dot size refers to the minimum value of the abundance of the three genes. **B** Transcriptional expression of 4-BP synthetic genes, based on OM-RGCv2_metaT dataset analysis. The dots represent the stations where all three genes are expressed and the dot size refers to the minimum expression level of the three genes. **C** The metagenomic assembled genomes (MAGs) containing all the three genes for 4-BP synthesis. (MAG data from Tully et al. 2018). **D** The prediction of the bacteria that contain 4-BP synthetic gene cluster and related gene alignments.

Our global microbiome data analysis showed that the three genes required for 4-BP synthesis (*bp608*, *bp609* and *bp610*) are widely distributed in the global oceans (Fig. 3A), and the metatranscriptomic data revealed that all three genes exert transcriptional activity in a wide range of environments (Fig. 3B). Indeed, based on a database containing 2,631 draft metagenomic assembled genomes (MAGs) from the global oceans [15], 13 MAGs containing all three of the genes were found to be widely distributed at 35 sampling sites in different seas (Fig. 3C, Table S2). They were affiliated with a diverse group of bacteria including: orders *Oceanospirillales* and novel_E1 in the *Gammaproteobacteria*; order *Anaerolineales* in the *Chloroflexota*; order *Flavobacteriales* in the *Bacteroidota*; order *Gemmatimonadales* in the *Gemmatimonadota*; and order *Acidimicrobiales* in the *Actinobacteria*.

We also found that 66 genomic sequences out of 2750 whole genome sequences possessed *bp608*, *bp609* and *bp610* and these genes were arranged in tandem in each genome (Fig. 3D, Table S3). These sequences belonged to 36 bacterial strains that were classified into eight genera: i.e., *Microbulbifer*, *Pseudoalteromonas*, *Hahella*, and *Marinomonas* in the *Gammaproteobacteria*; and *Hormoscilla*, *Symploca*, *Nodularia*, and *Leptolyngbya* in the *Cyanobacteriota*. It is also worth noting that genes that are upstream and downstream of the three 4-BP synthesis genes are distinct in different bacterial genomes. This suggests that there might be differences in the regulation of the gene functions that perform 4-BP synthesis among the various genera. Those cyanobacterial genomes (for example, *Symploca* genomes) that contain 4-BP-synthesizing gene clusters tend to have multiple copies of the *bp610* gene (encoding cytochrome P450), which suggests that the metabolic process involved in the synthesis of 4-BP is unique in *Cyanobacteriota*.

### 4-BP seriously interferes with algal photosynthetic physiology

**Fig. 4:**
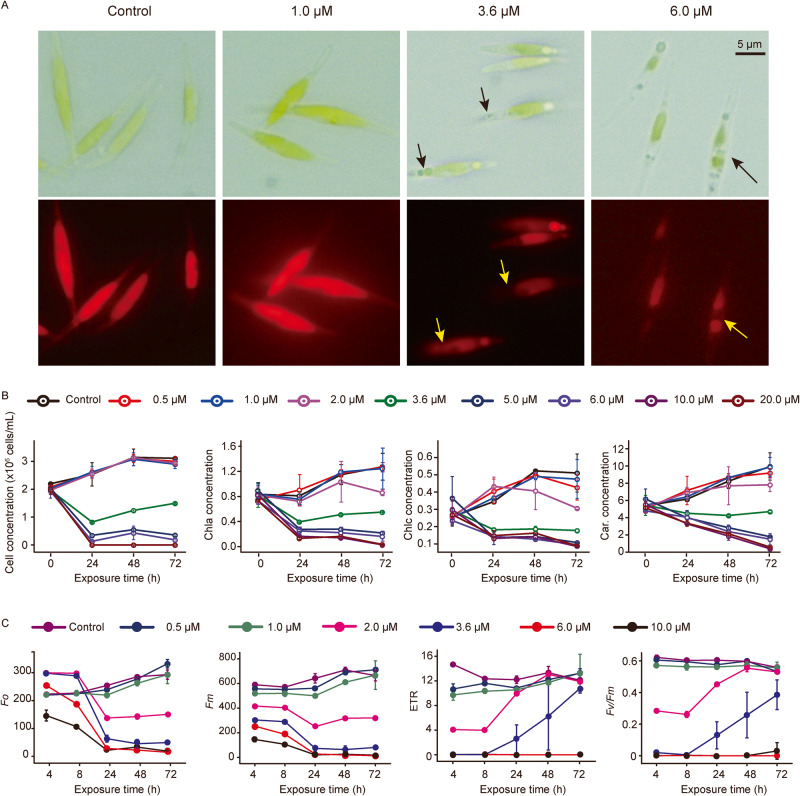
Morphology, amount of pigment and photophysiological response of *Phaeodactylum tricornutum* CCMP2561 to 4-BP. **A** Bright-field and fluorescence images of *Phaeodactylum tricornutum* CCMP2561 following treatment with 0, 1.0, 3.6 or 6 µM 4-BP for 72 h. The arrows indicate regions exhibiting a loss of pigment. **B** The number of cells and concentration of pigments (Chla, chlorophyll *a*; Chlc, chlorophyll *c*; and Car, carotenoids) in *Phaeodactylum tricornutum* CCMP2561 after treatment with 4-BP. **C** Quantification of various photosynthesis parameters in *Phaeodactylum tricornutum* CCMP2561 following treatment with 4-BP. Photosynthesis parameters measured include: the initial chlorophyll fluorescence (*Fo*), maximum chlorophyll fluorescence (*Fm*), electron transport rate (ETR) and maximum photosynthesis efficiency of photosystem (*Fv/Fm*).

The eukaryotic alga *Phaeodactylum tricornutum* CCMP2561 (hereafter abbreviated to *P. tricornutum*) was used as a model phytoplankton to explore the algicidal mechanism of 4-BP. The effective concentration 50 (EC_50_) value of 4-BP at 72 h based on algal abundance was 3.6 μM. Microscopic observations showed that 4-BP at 3.6 μM or 6.0 μM caused the algal cells to rupture and lose pigment (Fig. 4A). With regarded to specific photosynthetic pigments, 4-BP at ≥3.6 μM resulted in a decrease in the amount of chlorophyll *a*, *c*, and carotenoid with increasing exposure time (Fig. 4B). The extent of this inhibitory effect increased with the increase in 4-BP dosage. The *Fo* (initial chlorophyll fluorescence) and *Fm* (maximum chlorophyll fluorescence), widely used to determine the physiological status of phytoplankton, were also shown to be inhibited by 4-BP at ≥2.0 μM (Fig. 4C). In addition, the *Fv*/*Fm* (maximum photosynthesis efficiency of photosystem) and ETR (electron transport rate) both experienced an initial decrease with 4-BP at 2.0 μM or 3.6 μM, but then increased gradually with prolonged exposure. In contrast, at concentrations of 6 μM or 10 μM 4-BP, the *Fv/Fm* and ETR declined to 0 within the first 4 h and did not recover to the pre-treatment level even within 72 h (Fig. 4C). Overall, these results suggest that the pigments and photosynthetic systems of *P. tricornutum* are seriously affected by 4-BP.

### 4-BP significantly alters gene transcription in photosynthesis

4-BP strongly affected the basic metabolic processes of *P. tricornutum* by altering the expression of genes involved in cell redox homeostasis, photosynthesis, pigment synthesis, carbon metabolism, energy metabolism, and amino acid metabolism (Table S4, Figs. S2–6). Increasing the concentration of 4-BP exacerbated these transcriptional changes.

**Fig. 5:**
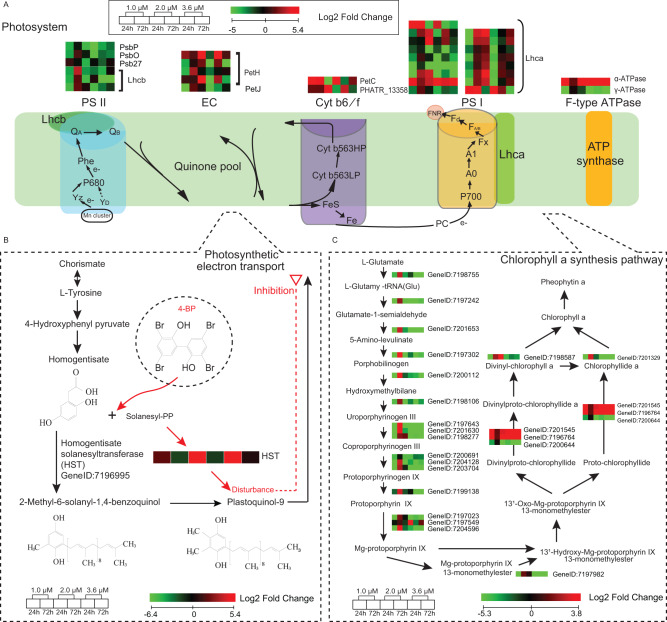
Effect of 4-BP on photosynthesis in *Phaeodactylum tricornutum* CCMP2561 at the transcriptional level. **A**–**C** Effect of 4-BP on the regulation of (**A**) the light reaction of photosynthesis, (**B**) plastoquinone-9 biosynthesis and (**C**) chlorophyll *a* synthesis.

With regards to the photosynthesis-related genes, exposure to 2.0 μM or 3.6 μM of 4-BP resulted in some genes encoding light-harvesting protein complexes (Lhcb) of photosystem II (PSII), as well as *PsbP*, *PsbO* and *Psb27* being down-regulated (Fig. 5A). However, at the same 4-BP concentrations, the genes encoding Cytochrome *bf/6* complex subunit (Cyt *b*_*6*_*/f*) protein PetC were initially down-regulated (i.e., within 24 h), but then the level of expression had recovered within 72 h. With regards to photosystem I (PSI), only a few genes encoding Lhca responded to 4-BP. Moreover, the *PetH* and *PetJ* genes of the photosynthetic electron transport system were mostly up-regulated by 2.0 μM or 3.6 μM 4-BP within 24 h but down-regulated within 72 h. Regarding the chlorophyll *a* synthesis pathway, 1.0 μM 4-BP resulted in an initial (i.e., within 24 h) down-regulation of genes involved in the biosynthesis of Mg-protoporphyrin IX 13-monomethylester from L-glutamate, but thus their expression was re-upregulated by 72 h (Fig. 5C). However, at high concentrations (2.0 μM or 3.6 μM) of 4-BP, the down-regulation of these genes continued even at 72 h (Fig. 5C). These results suggest that 4-BP might interfere with intermediate processes in the algal photosynthetic chain, causing upstream and downstream genes in photosynthesis to respond differently.

### 4-BP limits the biosynthesis of plastoquinone-9 in the photosynthetic electron transport

The inhibition of algal *Fv/Fm* and ETR indicated that 4-BP might affect the photosynthetic electron transport chain (pETC). This was particularly obvious at concentrations of 6 μM or 10 μM 4-BP when the *Fv/Fm* and the ETR became zero (Fig. 4C), suggesting that the pETC was totally blocked. Since these PSII fluorescence parameters (i.e., *Fv/Fm* and ETR) are directly related to the reduction and oxidation kinetics of plastoquinone in the reaction center, we propose that 4-BP might have an inhibitory effect on the PSII light reaction center, especially on plastoquinone-9. There is a key enzyme in the biosynthetic pathway of plastoquinone-9, called homogentisate solanesyltransferase (HST), which catalyzes the condensation of homogentisate with all-trans-nonaphenyl diphosphate solanesyl diphosphate, to form 2-demethylplastoquinol-9, and this branch-point compound directly leads to the biosynthesis of the plastoquinone-9 [11] (Fig. 5B). Here, we showed that at concentrations of 2.0 μM and 3.6 μM 4-BP, the HST gene was up-regulated by up to 28- to 41-fold at 24 h, but it returned to normal levels by 72 h (Fig. 5B). No significant (*p* > 0.05) differences in the HST gene were found following treatments with 1.0 μM 4-BP. These HST expression patterns corresponded well to the ETR and *Fv/Fm* pattern, suggesting that the impaired algal photosynthesis and cell death observed in *P. tricornutum* following treatment with 4-BP might be directly due to the down-regulation of HST.

**Fig. 6:**
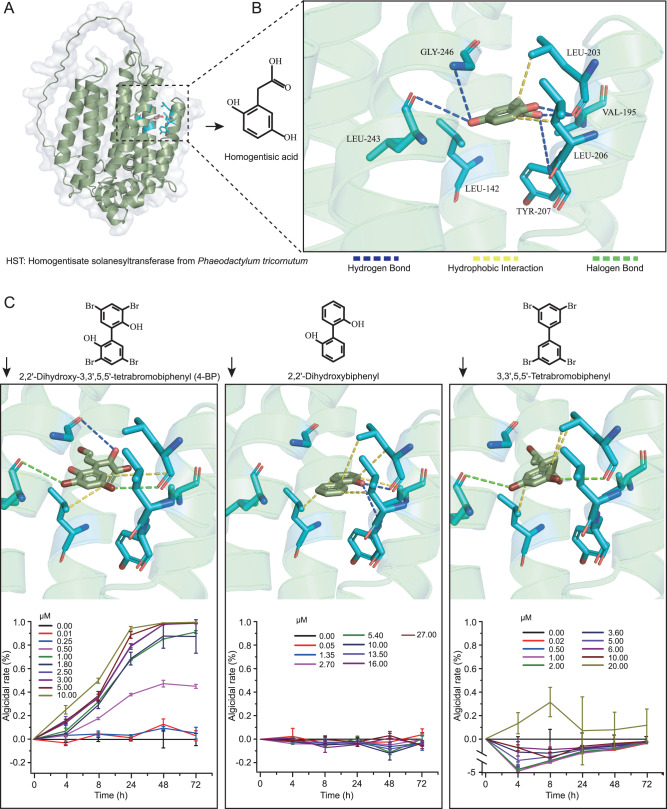
Structure prediction of homogentisate solanesyltransferase (HST) from *Phaeodactylum tricornutum* CCMP2561 and its substrate docking. **A** The three-dimensional structure of the HST sequence was constructed using ColabFold; (**B**) Docking studies (with AutoDock) showed the binding of homogentisic acid to HST. Amino acids at the HST active site are shown such that: LEU leucine, GLY glycine, VAL valine, and TYR tyrosine. Homogentisic acid docked inside the active site and linked to these amino acids with different interactions; (**C**) The binding of three structurally similar molecules (i.e., 4-BP, 2,2’-dihydroxybiphenyl and 3,3’,5,5’-tetrabromobiphenyl) to the HST are shown along with their algicidal effect on *Phaeodactylum tricornutum* CCMP2561, when tested at different concentrations.

For demonstrating the effect of 4-BP on HST function, we heterologously expressed HST from *P. tricornutum* and performed in vitro enzymatic activity assays. Several efforts failed to obtain enzymatically active HST from this diatom, perhaps because the protein has an eight-transmembrane structure, which makes it difficult to form intact proteins following heterologous expression (Fig. 6A, Supplementary Fig. S7). Then, using another method, that is HST structure prediction and substrate docking analysis, we revealed that 4-BP has the same active site as homogentisic acid, i.e., the HST substrate. The region formed by LEU_203, TYR_207, LEU_243, and GLY_246 of HST is a potential active site for homogentisic acid, and the amino acids interact with homogentisic acid through hydrogen and hydrophobic bonds (Supplementary Table S5). We showed that 4-BP could interact with three of these amino acids (i.e., LEU_203, LEU_243 and GLY_246) through two halogen bonds, two hydrophobic bonds and one hydrogen bond, and has comparable site competition to homogentisic acid. The results indicate that 4-BP can compete for the HST active site of homogentisic acid and thus inhibit the subsequent biosynthesis of plastoquinone-9.

To further verify the target sites of 4-BP, we performed the same analysis using two molecules similar in structure to 4-BP, namely 2,2’-dihydroxybiphenyl and 3,3’,5,5’-tetrabromobiphenyl. Although they also have the potential to interact at the above sites, they both only interact with two of the amino acids. Thus, 2, 2’-dihydroxybiphenyl interacts with LEU_203 and TYR_207 through hydrophobic and hydrogen bonds, and 3,3’,5,5’ -tetrabromobiphenyl interacts with LEU_203 and LEU_243 through hydrophobic and halogen bonds. We suggest that it is this weak effect of 2,2’-dihydroxybiphenyl and 3,3’,5,5’-tetrabromobiphenyl on the homogentisic acid binding site, which makes them ineffective algicides (Fig. 6B). Thess data also indirectly confirm that 4-BP can compete for the HST active site of homogentisic acid.

### Introduction of 4-BP alters the phytoplankton community structure of a simulated algal bloom

We incubated 200-μm-prefiltered natural seawater (supplemented with nutrients rich in inorganic nitrogen and phosphorus) under natural conditions, to obtain a simulated algal bloom. After 5 days of incubation, a brown algal bloom appeared with a chlorophyll-*a* concentration of 42.6 mg/m^3^ (Supplementary Fig. S8). *Dinoflagellata* (45%), *Cryptophyta* (30%) and *Bacillariophyta* (26%) became the dominant phytoplankton in the algal bloom based on 18 S rRNA gene analysis (Supplementary Fig. S9). In terms of chlorophyll *a* concentration, 4-BP suppressed algal blooms and had a dose effect. For the phytoplankton community structure, the introduction of 4-BP at a low concentration (0.05 μM) resulted in marked changes and these changes became more pronounced as the concentration of 4-BP was increased. At 0.05 μM 4-BP, the relative abundance of the *Bacillariophyta* showed a marked decline. In contrast, the *Cryptophyta* increased instead (Supplementary Fig. S9A, B). This change became more obvious at the higher concentrations of 4-BP (i.e., 0.5 μM), such that *Cryptophyta* became the most dominant phylum, with a relative abundance of above 95%. In addition, the relative abundance of *Dinoflagellata* and *Haptophyta* also decreased with the increase of 4-BP concentrations. At the genus level, with 0.05 μM or 0.5 μM 4-BP treatments, the relative abundance of the genera *Protoperidinium* (affiliated with *Dinoflagellata*) and *Thalassiosira* (affiliated with *Bacillariophyta*) significantly decreased, whereas the relative abundance of the genera *Cryptomonadales*_g and *Rhodomonas* (affiliated with *Cryptophyta*) significantly increased (Mann-Whitney test, *p* ≤ 0.05, Supplementary Fig. S9C). The varied susceptibility of different phytoplankton taxa to 4-BP might be related to their unique characteristics in trophic metabolism in addition to photoautotrophy.

## Discussion

Here, we showed that a marine member of the *Gammaproteobacteria* exhibits algicidal activity against a diverse array of phytoplankton via the production of an extracellular compound. In fact, similar algicidal phenomena have been observed in a variety of bacterial species, including other strains in the genus *Microbulbifer* [8, 16]. However, the algicidal compounds are currently great enigmas for us as the vast majority of them are unknown. Here, we successfully isolated and identified a new algicidal compound (i.e., 4-BP) produced by bacteria and revealed for the first time that it can cause mortality of various phytoplankton, including diatoms, chlorophyta, and cyanophytoa.

We also obtained a naturally differentiated or mutant strain, *Microbulbifer* sp. TB12003. This has the same 16 S rRNA gene sequence and a highly similar genome (i.e., 99.95% identical) as RZ01, but it does not have any algicidal activity. Through whole-genome sequencing and comparative genomic analysis of the two strains, we predicted the genes in RZ01 that synthesize 4-BP. Heterologous expression experiments verified that the co-expression of three genes in a gene cluster (encoding chorismate lyase, flavin-dependent halogenase and cytochrome P450 enzyme) successfully synthesized 4-BP in the presence of chorismate and bromide ions (Fig. 2C). Whether 4-BP can also be synthesized by these genes in other bacteria or not, likely depends on other factors. For example, strain TB12003 also contains these three genes, but a single base loss in an upstream gene (which encodes an HTH-type transcriptional activator), prevents the synthesis of 4-BP. The HTH-type transcriptional activator is adjacent to the phospho-2-dehydro-3-deoxyheptonate aldolase gene (*bp607*; Fig. 2B), and the enzyme it encodes, catalyzes the first step of the shikimate pathway reaction responsible for the synthesis of chorismate. We speculate that the HTH-type transcriptional activator might lead to changes in the chorismate content by affecting the transcriptional level of phospho-2-dehydro-3-deoxyheptonate aldolase, and this, in turn, might regulate the conversion of chorismate to 4-BP. The disruption of the HTH-type transcriptional activator in the mutant strain might therefore result in a loss of this regulatory process.

We also found that another cultured *Microbulbifer* bacterial strain (*Microbulbifer rhizosphaerae* CECT 8799) and several other taxa affiliated with the *Gammaproteobacteria*, including *Pseudoalteromonas luteoviolacea* (15 strains) and *Marinomonas mediterranea* MMB-1, possess the 4-BP-synthesizing gene cluster. It was reported that *Pseudoalteromonas luteoviolacea* had the broadest anti-algal activity among 10 *Pseudomonas* species tested [17]. Furthermore, close relatives of *Marinomonas mediterranea* MMB-1 have previously been detected as growth-limiting bacteria of the perpetrator species of *Chattonella* blooms [18]. In these previous reports, the inhibitory mechanism of the bacteria was not very clear, but we speculate that 4-BP might have been involved in antagonizing the algae. Some cyanobacteria also have the potential to synthesize 4-BP (Fig. 3D). However, the arrangement of 4-BP-related gene clusters and the upstream and downstream genes are very different in cyanobacteria from those in bacteria, and so this suggests that cyanobacteria may have unique regulation mechanisms for 4-BP synthesis.

Analysis of MAGs from the global ocean microbiome data, revealed that the three 4-BP synthesis genes together are also present in other cosmopolitan marine bacterial taxa (including the *Chloroflexota*, *Bacteroidota*, *Gemmatimonadota*, and *Actinobacteria*), and they are widespread in the global oceans. 4-BP was also found in the brown algae *Sargassum* sp., marine sponges and bull sharks, but it is unclear whether 4-BP is synthesized by these organisms themselves or by their symbiotic or epiphytic microbes [19–21]. Our findings suggest that there are many other bacterial taxa in the ocean that synthesize 4-BP, but these are still to be identified. However, we propose that 4-BP is likely to be a widely used chemical tool in mediating the antagonistic relationship between bacteria and phytoplankton in the ocean.

4-BP is a member of the polybrominated aromatic organic compounds. In general, aromatic organic compounds do not readily degrade, and so 4-BP might exist in the seawater for a long time. Therefore, 4-BP might accumulate to a high concentration in locations where there is a relatively closed marine environment rich in organic matter, thereby mediating the inhibition of algae by bacteria. At certain concentrations, 4-BP affects most of the metabolic processes in algae, especially photosynthesis. Photosynthesis is often the target of antagonistic bacteria or toxic molecules, for example, prodigiosin (from bacteria) inhibits the efficiency of photosynthesis in the harmful alga species, *Phaeocystis globose* [22]. The amount of photosynthetic pigments is an important indicator of the growth status and biomass of phytoplankton [23]. We showed that 4-BP had serious toxic effects on *P. tricornutum*, due to the reduction in pigment content and fluorescence intensity. These toxic effects are also manifested at the transcriptomic level, such that most of the genes in the chlorophyll *a* synthesis pathway, (especially from L-glutamate to Mg-protoporphyrin IX 13-monomethylester), were inhibited by 4-BP (when used at 2.0 μM and 3.6 μM) at 24 h and 72 h (Fig. 5C). However, the transcriptional changes of most genes in this pathway showed a consistent trend, suggesting that 4-BP might not impair the intermediate steps of chlorophyll *a* synthesis. Therefore, we propose that 4-BP might affect other photosynthetic pathways other than the chlorophyll *a* synthesis pathway.

We did find that 4-BP caused the differential expression of genes encoding proteins that function successively in the photosynthetic chain. After exposure to higher concentrations (i.e., 2.0 μM or 3.6 μM) of 4-BP for 24 h, *PsbP*, *PsbO*, and *Lhcb* were down-regulated, while *PetC*, *PetH*, and *Lhca* were mostly up-regulated. This suggests that 4-BP might interfere with the intermediate process of photosynthetic electron transfer. Previous studies have also reported that the electron transport chain of photosynthesis can be affected by external molecules. For example, treatment of the diatom *Thalassiosira pseudonana* with the herbicide thiobencarb, resulted in a downregulation of PetC protein [24], suggesting that this chemical might target Cyt *b*_*6*_*/f*. In addition, the photosynthesis inhibitor DBMIB, also targets Cyt *b*_*6*_*/f* to block electron transport chain [25]. Here, we speculate that plastoquinone might be a potential target of 4-BP. This is because: (1) 4-BP significantly inhibited the *Fv/Fm* and ETR, which are closely related to plastoquinone; (2) 4-BP induced a > 20-fold upregulation of the *hst* genes, which are known to be involved in plastoquinone-9 synthesis; and (3) The HST response to 4-BP concentration and exposure time were consistent with changes in algal physiology. HST is an important enzyme that mediates the synthesis of the plastoquinone-9 precursor, and the effect of 4-BP on HST likely resulted in the inhibition of plastoquinone-9 synthesis. To test our hypothesis, we performed structure prediction and substrate docking analysis of HST and revealed that 4-BP had the same active site as the natural HST substrate, homogentisic acid. 4-BP competitively occupied three of the four amino acids at the homogentisic acid-HST docking site, but with different interaction forces. This might therefore be the mechanism by which 4-BP causes the abnormal expression of HST. Therefore, we propose that 4-BP is a pETC inhibitor that can inhibit or kill algae by interfering with the synthesis of plastoquinone-9, thereby disrupting electron transport in the photosynthetic light reactions.

The effect of 4-BP on photosynthetic electron transport might also cascade to other key metabolic pathways, such as the Calvin cycle, a downstream process of the photosynthetic light reactions and the only carbon fixation pathway in phytoplankton (Supplementary Fig. S2). In addition, the photosynthetic electron transport chain is known to be a constant source of reactive oxygen species (ROS) [26], so it was interesting to note that treatment of *P. tricornutum* with 4-BP induced an increase in ROS (Supplementary Fig. S6). This increase in ROS along with the identification of differentially expressed genes involved in redox hemostasis indicate that 4-BP caused oxidative stress in the algal cells (Supplementary Table S4). Surplus ROS can react with almost all the components of living cells, damaging lipids, pigments, proteins, nucleic acids, and organelles, eventually leading to cell death [27–29]. ROS can also act as a second messenger that regulates gene expression of the nucleus and organelles [28, 30]. Collectively, 4-BP might interfere with photosynthetic electron transfer and subsequently alter algal physiology through a cascade of reactions, even leading to cell death when at high concentrations.

In general, phytoplankton nourish the surrounding heterotrophic microbes by releasing dissolved organic carbon, and nutrient cycling among them supports their long-term survival especially in oligotrophic environments [6]. However, environmental changes such as eutrophication can lead to the abnormal growth of phytoplankton. This is accompanied by the rapid growth and community succession of heterotrophic bacteria and thus it changes the relationship between the phytoplankton and bacteria. In the later stages of an algal bloom, bacteria that have an antagonistic relationship with the phytoplankton often greatly increase in abundance through rapid proliferation. Correspondingly, the algicidal substances released by these bacteria quickly accumulate to a relatively high concentration that can exert an algicidal effect, and thus lead to the decline of the algal bloom [4]. From this perspective, algicidal bacteria act as important regulators of the marine ecosystem. Here, we experimentally simulated an algal bloom scenario under eutrophication and obtained a phytoplankton community dominated by the *Dinoflagellata, Cryptophyta and Bacillariophyta* (Supplementary Fig. S9). We revealed that 4-BP significantly inhibited the *Bacillariophyta* and *Dinoflagellata* members (Mann-Whitney test, *p* ≤ 0.05) and altered the structure of the phytoplankton community, even at low concentrations (i.e., ≤0.5 μM). However, the sensitivity of the different phytoplankton taxa to 4-BP was obviously different. *Protoperidinium* (affiliated with *Dinoflagellata*) and *Skeletonema* (*Bacillariophyta*) appeared to be more sensitive to 4-BP exposure than the *Cryptophyta*, resulting in a clear decrease in their relative abundance when treated with 4-BP. In contrast, genera *Cryptomonadales*_g and *Rhodomonas* belonging to *Cryptophyta* have less susceptibility to 4-BP, and their relative abundance increased instead. The *Dinoflagellata* and *Bacillariophyta* are ubiquitous photosynthetic eukaryotes and they are a major causative group of algal blooms in the ocean [31, 32]. Nonetheless, not all members of the *Dinoflagellata* and *Bacillariophyta* were completely inhibited by 4-BP even when exposed to high concentrations, perhaps because some are facultative heterotrophs [33]. In contrast, members of the *Cryptophyta*, are better able to grow mixotrophically than the *Bacillariophyta* [34]. In addition, the relative abundance of a *Cryptophyta* member *Goniomonas*, which lacks plastid, did not significantly correspond to 4-BP concentration, providing indirect evidence for the inference that 4-BP affects photosynthesis [35]. Moreover, *Cryptophyta* contain phycobiliproteins or alloxanthin [36], which might help to reduce the oxidative stress induced by algicidal substances and weaken the susceptibility of *Cryptophyta* to 4-BP [37, 38]. We admit that the phytoplankton community composition obtained using molecular techniques is often inconsistent when compared with morphological observations, and should introduce some biases in this study; however, molecular techniques can also identify those algal species that cannot be detected by morphological observations alone [39, 40]. In addition, it is worth noting that these results still require further validation with larger sample sizes. Here, we propose that the algicidal bacteria that synthesize 4-BP might play an important role in the decline of algal blooms by inhibiting photosynthesis and remodeling the phytoplankton community structure. As the bacteria that can synthesize 4-BP are diverse and the 4-BP synthesis genes are widely distributed in the global oceans, this small molecule might prove to be a widely used tool for algicidal bacteria to combat the overgrowth of phytoplankton. It is also worth noting that 4-BP synthesis in bacteria is transcriptionally regulated and closely related to the growth state of the bacterial cells and the external environment, and the dynamic interactions between 4-BP synthesizing bacteria and phytoplankton deserve further study in the future.

## Methods and materials

### Bacterial strains and cultivation

Two strains, *Microbulbifer* sp. RZ01 and sp. TB12003, isolated from the coastal environment of Qingdao, China, were used in this study (Supplementary Text and Figs. S10, 11). Complete genome sequencing (detailed methods are described below) revealed that these two strains shared the same 16 S rRNA gene and have high genomic similarity (99.95% average nucleotide identity). However, they have different effects on algal growth, such that strain RZ01 inhibits phytoplankton growth, whereas strain TB12003 does not. Here, for the purpose of this study, we regarded RZ01 as being a wild strain, and TB12003 as a natural mutant strain that has lost its algae inhibitory activity. We speculated that the few genes that are different in TB12003 might be vital for the algicidal effects of RZ01. Both strains were routinely grown in 2216E medium at 28 °C for subsequent experiments.

### Detection of the bacterial algicidal effect against different phytoplankton

Eleven algal strains affiliated with different taxa were employed to assess the spectrum of anti-algal activities of *Microbulbifer* sp. RZ01 (Supplementary Table S1). All the algal strains were maintained in f/2 medium [41] at 20 °C in a 12 h:12 h light-dark cycle at 65 μmol photons m^−2^ s^−1^, except for *Prochlorococcus marinus* CCMP2389 (maintained in Pro99 medium at 20 °C) [42] and *Synechococcus* sp. PCC7002 (maintained in A^+^ medium at 25 °C) [43]. Strain RZ01 was inoculated into 2216E medium and grown for 60 h at 28 °C. Triplicate co-cultures were then established by inoculating the bacterial culture into exponentially growing algal cultures at concentrations of 5 and 10% (v/v). Control cultures (axenic) were supplemented with the corresponding volume of 2216E medium. The algicidal rate was calculated from the changes in fluorescence intensity measured daily during co-cultivation at an excitation and emission wavelengths of 440 nm and 680 nm, respectively. It was defined as follows: *Algicidal rate* = (FC − FT) / FC × 100%, where FT and FC represent the fluorescence intensity of the algal culture with and without bacteria, respectively. All experiments were performed in triplicate.

### Purification and identification of algicidal compounds

Bacterial algicidal patterns and the properties of the algicidal substances were analyzed. The latter included determining the thermal and pH stability of the algicidal substances, as well as the range of molecular sizes (Supplementary Methods). The algicidal compounds were then isolated and purified. In brief, the RZ01 culture was grown to the decline phase, after which the supernatant was collected by centrifugation at 8000 rpm for 10 min and then mixed with an equal volume of ethyl acetate. The substances in the cell pellet were extracted sequentially with methanol (MeOH) and ethyl acetate. The two ethyl acetate extracts were combined and rotary evaporated to obtain a dried powder. This crude extract was then dissolved in MeOH for further purification. Crude extracts were separated by MPLC using a gradient elution of MeOH–H_2_O (30–100%). The yielded fractions were then added one by one to the algal cultures to test their individual algicidal effects. The active fractions (i.e., those showing algicidal effects) were further separated by semipreparative HPLC, eluted with CH_3_CN–H_2_O (70%) and/or MeOH–H_2_O (34–100%). The purified compounds were collected and their molecular weight and structure were determined using a mass spectrometer and NMR including ^1^H-NMR, ^13^C-NMR and two-dimensional ^1^H-^13^C HMBC [44]. The algicidal effect of the purified algicidal compound (i.e., 3,3’,5,5’-tetrabromo-2,2’-biphenyldiol, 4-BP) was then evaluated according to changes in fluorescence intensity of the exponentially growing *Synechococcus* sp. PCC7002, as described above.

### Whole genome sequencing and comparison for prediction of key genes involved in algaecide synthesis

Genomic DNA was extracted from strains RZ01 and TB12003 using a DNA extraction kit (TIANGEN, Beijing, China). The high-quality DNA obtained was sequenced using a HiSeq x-Ten (Illumina) and PacBio RSII long-reading platforms at Novogene (Beijing, China). Approximately 90,000 clean reads, with an average length of ~8000 nucleotides, were obtained from the PacBio platform and assembled with Canu [45]. In addition, Illumina reads were mapped to the polished PacBio assembly to correct insertions and deletions with bowtie2 [46] and pilon [47], yielding the complete genomes. Gene prediction was performed by Prokka version 1.14.6 [48] with default parameters and functional annotation was done with KofamScan version 1.3.0 (E-value ≤ 1 e^−5^) [49] and eggNOG 5.0 (E-value ≤ 1 e^−5^) [50]. A comparative genomic analysis of strains RZ01 and TB12003 was performed using Mauve [51], to determine the genes unique to each. Circular genome map was visualized using the program DNAPlotter [52]. A detailed functional analysis of the unique genes of the two strains and the gene clusters adjacent to these unique genes was then performed to predict the key genes involved in the synthesis of the algicidal substance, 4-BP.

### Cloning, protein expression, and functional validation of the algicide-related genes

Predicted algicide-related genes *bp608*, *bp609* and *bp610* were amplified by PCR from RZ01 genomic DNA using the geneF-NdeI and geneR-SpeI-SalI primers (Supplementary Table S6), and inserted into the pET28a(+) expression vector via NdeI and SalI to construct pET28a-*bp608*, *-bp609* and *-bp610*. The SalI-XbaI fragment of *bp609* from pET28a-*bp609* was inserted into the SalI and SpeI sites of PET28a-*bp608* to construct pET28a-*bp608*-*bp609*. The SalI-XbaI fragment of *bp610* from pET28a-*bp610* was then inserted into the SalI and SpeI sites of pET28a-*bp608*-*bp609* to construct pET28a-*bp608*-*609*-*610*. These recombinant vectors were introduced into competent *E. coli* BL21 (DE3) for protein expression. Protein expression was induced by the addition of 0.5 mM isopropyl *β*-D-1-thiogalactopyranoside (IPTG) and cultures were grown overnight at 18 °C. Cells were collected by centrifugation at 8000 rpm for 10 min at 4 °C and resuspended in the buffer (50 mM Tris-HCl, pH 7.5, 500 mM NaCl and 10% glycerol). The cell suspension was then sonicated and centrifuged at 15,000 g for 30 min at 4 °C. The whole cell lysates, supernatants and precipitates were analyzed by SDS-PAGE against the target proteins. The supernatant containing the target protein was applied to a Ni-NTA column equilibrated in the above-mentioned buffer. The protein was then eluted using a linear gradient of imidazole from 0 to 500 mM. The protein was then dialyzed in 50 mM Tris-HCl (pH 8.0), 500 mM NaCl and 10% glycerol, and stored at −80 °C.

The enzyme activity of 4-hydroxybenzoate acid synthase (BP609) was verified by measuring the presence of the target product, p-hydroxybenzoic acid, in the reaction mixture containing the supernatant of the *E. coli* cultures expressing *bp609* and the substrate of the enzyme (chorismate) using LC-MS. To measure the enzyme activity of ferredoxin-NADP reductase (BP608), the reaction contained *E. coli* cultures expressing *bp608*, the substrate p-hydroxybenzoic acid, and KBr. After 24-h reaction and ethyl acetate extraction, the target product, 2,4-dibromophenol, was measured using LC-MS. The activity of cytochrome-P450 (BP610), was not tested directly by measuring the reaction between the enzyme and its substrate (2,4-dibromophenol), due to the instability of the latter. However, a tandem enzymatic reaction was used, which utilized BP608 as well as BP610, along with a substrate of the former, i.e., p-hydroxybenzoic acid or 3, 4-dihydroxybenzoic acid. The resulting target product of BP610 (4-BP) was measured using LC-MS.

### The distribution of algicide-related genes in global oceanic microbes

The global distribution of the algicide-related genes (*bp608*, *bp609* and *bp610*) was determined by querying their protein sequences against the Tara Oceans Microbiome reference gene catalog with arctic data (OM-RGCv2-metagenomes and -metatranscriptomes) [53], with a threshold E-value ≤ 1 e^−10^. These protein sequences were also queried against each annotated gene protein sets of 2631 metagenomic assembled genomes (MAGs) derived from a previous study of the Tara Ocean database using Blastp (E-value ≤ 1 e^−5^) with default settings [15] and MAGs containing the three genes were manually screened. To identify which microbes contain the *bp608*-*609*-*610* gene cluster, BP608, BP609 and BP610 protein sequences were queried against the NCBI nr database, and the genomes containing at least one of the three genes were obtained. A total of 2750 whole genomes involving 26 bacterial taxa were obtained. From these, the bacteria with the three genes adjacent and sequentially arranged in their genome were screened and considered as potential producers of 4-BP; the algicide-related gene arrangement was visualized by chiplot (https://www.chiplot.online/index.html).

### Effect of 4-BP on algal physiology

*P. tricornutum*, a widely used model diatom, was selected as to test the effect of 4-BP on algal physiology. Fluorescence intensity, chlorophyll *a* concentration, and photosynthesis efficiency were determined after the addition of 4-BP (at final concentrations of 0, 0.5, 1.0, 2.0, 3.6, 5.0, 6.0, 10.0, and 20 μM) into the exponentially growing *P. tricornutum*. These cultures were incubated for 72 h at 20 °C in a 12 h: 12 h light-dark cycle with a photosynthetic photon flux density of 65 μmol photons m^−2^ s^−1^. The concentration of chlorophyll *a*, *c*, and carotenoids was determined according to the previous methods [54]. Photosynthetic parameters including *Fo*, *Fm*, *Fv/Fm*, and ETR were measured using a pulse amplitude-modulated fluorometer (Water-PAM fluorometer, Walz, Germany) after the samples were dark-adapted for 20 min. ROS production was measured using the modified method described elsewhere [55]. The half maximal EC50 at 72 h was calculated using a sigmoidal dose-response curve according to the amount of chlorophyll *a* and cell abundance.

### Analysis of algal transcriptome response to algicide

4-BP was added to exponentially growing *P. tricornutum* cultures at final concentrations of 1.0, 2.0 or 3.6 μM. *P. tricornutum* cultured in the absence of 4-BP served as a control. The transcriptional responses of the algal cells were detected at 24 h and 72 h. The algal cells were harvested by centrifugation at 5,000 g for 15 min, immediately frozen in liquid nitrogen, and stored at −80 °C until RNA extraction. The preserved cells were then mechanically lysed using previous methods [56]. Total RNA was isolated using TRIzol (Invitrogen, Carlsbad, CA), and purified using Mini Spin Columns from RNeasy Mini Kits (Qiagen, Valencia, CA). The integrity and quality of the RNA were then checked using an Agilent 2100 Bioanalyzer (Agilent, Santa Clara, CA). A cDNA library for the subsequent cluster generation was prepared using the reagents provided in the TruSeq RNA sample preparation kit (RS-122-2001, Illumina Inc., San Diego, CA). Sequencing was accomplished by a commercial service (Oebiotech, Shanghai, China) using a HiSeq x-Ten. The raw data were then processed using NGS QC Toolkit [57] and reads of low quality or containing poly-N were removed (cutoff quality value: 20; cutoff read length for high quality: 70%). The resulting clean reads were then mapped to the reference genome (NCBI accession number: GCF_000150955.2_ASM15095v2) using hisat2 [58]. The FPKM (fragments per kb per million reads) value of each gene was then calculated using cufflinks [59, 60], and differentially expressed genes (DEGs) were identified using DESeq [61]. A fold change >2 (*p* < 0.05) was set as the threshold for significantly differential expression. Gene Ontology enrichment analysis and Kyoto Encyclopedia of Genes and Genomes (KEGG) pathway [62] enrichment analysis of the DEGs were both performed, using R based on the hypergeometric distribution. Corrected *p*-value < 0.05 cutoff was used to determine significance.

### In silico verification of the action pathway of algicidal substance

A partial sequence of the *P. tricornutum hst* gene was obtained from genome data (NCBI Reference Sequence: XM_002178060.1) and its complete sequence was determined using the Takara SMARTer RACE 5’/3’ kit (TaKaRa). The resulting full-length ORF of HST was 1164 bp in length with the sequence for the putative transit peptide of 22 amino acids (Supplementary Results). The three-dimensional structure of the HST sequence was constructed using ColabFold [63], and homogentisic acid and 4-BP were docked to HST using AutoDock [64]. To ensure the accuracy of our docking prediction, two other molecules that are structurally similar to 4-BP (i.e., 2,2’-dihydroxybiphenyl and 3,3’,5,5’-tetrabromobiphenyl), were used as controls, and their docking position with HST was established, as described above for 4-BP. All of the structures were visualized using PyMOL (http://www.pymol.org). In addition, the algicidal effect of these three structurally similar molecules was investigated to analyze the potential site(s) of 4-BP action on HST. They were added to exponentially growing *P. tricornutum* cultures at different concentrations and the growth dynamics of the diatom were examined. As 4-BP and 2,2’-dihydroxybiphenyl are water soluble, *P. tricornutum* cultured in the absence of these compounds, was used as a control. In contrast, 3,3’,5,5’-tetrabromobiphenyl is water-insoluble, and so in this case, *P. tricornutum* incubated with the same volume of hexane as 3,3’,5,5’-tetrabromobiphenyl, was used as a solvent control.

### Analysis of the response of the natural seawater phytoplankton communities to the algicidal substance

Natural seawater samples were collected from the coast of Qingdao, China in October 2022. Seawater was pre-filtered through a 200-μm pore size mesh to remove most of the large predators of phytoplankton. Final concentrations of 441 μM NaNO_3_ and 16.25 μM KH_2_PO_4_ were added to 1.5 L of the filtered seawater in 2-L flasks, to ensure that the phytoplankton communities would grow under eutrophic conditions. The water was then maintained under natural light/dark conditions for 5 days. 4-BP at three concentrations (0.005, 0.05 or 0.5 μM) was added to the pre-cultured seawater, and one flask without 4-BP was used as the control group. Each group had three replicates. The bottles were incubated at room temperature (20 °C-25 °C) for 3 days in the laboratory under natural lighting conditions. Subsamples at 1st and 3rd days were collected from each bottle for analysis of the changes in phytoplankton community structure and chlorophyll a concentration.

Each 500-ml subsample was filtered through a 2.0-μm pore-size polycarbonate membrane, and RNA was extracted from the membrane using an E.Z.N.A Soil RNA mini kit (Omega Bio-Tek, Georgia, USA). The RNA samples were then reverse transcribed into cDNA using the SuperScript First-Strand synthesis system with random hexamers for RT-PCR (Invitrogen, USA). PCR was performed with the following pair of 18 S rRNA gene primers, SSU0817F and 1196 R [65]. The PCR reaction system was as follows: 4 μl 5 × FastPfu buffer, 2 μL dNTPs (2.5 mM), 0.2 μL BSA, 0.8 μL each of forward and reverse primer (5 μM), 0.4 μL TransStart Fastpfu DNA Polymerase, 10 ng template DNA, and added double-distilled water to 20 μL. These systems were run at 95 °C for 3 min; 35 cycles at 95 °C for 30 s, 55 °C for 30 s, 72 °C for 45 s; at 72 °C for 10 min. The PCR amplicons were sequenced on a HiSeq2500 PE250 platform (Illumina) at Shanghai Majorbio Bio-Pharm Technology Co., Ltd. Paired-end reads were merged using flash v1.2.11 [66]. Primer removal and quality filtering were performed using the vsearch --fastx_filter with the fastq_maxee_rate set to 0.01. Unique sequence reads were found via the “vsearch --derep_fulllength” script (--minuniquesize 8). Denoise was performed with unoise3 in the usearch to generate amplicon sequence variants (ASVs). The ASV table was created using the vsearch --usearch_global. Taxonomy was assigned to the ASVs via the PR^2^ database and “vsearch –sintax” script (--sintax_cutoff 0.6) [67]. The feature table was rarefied with the vegan R package (http://www.r-project.org/). The eukaryotic phytoplankton reads (27,549 reads) were further selected for phytoplankton community structure analysis. The Mann-Whitney U-test was performed to analyse statistical differences between the control and treatment groups. In addition, chlorophyll *a* concentration was determined according to the reported methods [54].

### Supplementary information

Supporting Information

Supplementary table 1

Supplementary table 2

Supplementary table 3

Supplementary table 4

Supplementary table 5

Supplementary table 6

## Data Availability

The sequence data for *Microbulbifer* strains RZ01 and TB12003 were deposited in GenBank under accession codes CP116898 and CP116899, respectively. Sequence data for the *P. tricornutum* transcriptome were deposited into the NCBI Sequence Read Archive under accession numbers SRR23258135-SRR23258158, and the 18 S rRNA gene sequence data were deposited in the NCBI Sequence Read Archive under accession numbers SRR23250035-SRR23250049. The nucleic acid sequence of the homogentisate solanesyltransferase gene from *P. tricornutum* CCMP2561 and its corresponding amino acid sequence are available in the Supplementary Material.
