# Supplementary material for: Plastoquinone synthesis inhibition by tetrabromo biphenyldiol as a widespread algicidal mechanism of marine bacteria
Source: ISME J. 2023 Sep 8;17(11):1979–92. doi: 10.1038/s41396-023-01510-0 (PMC10579414; doi:10.1038/s41396-023-01510-0)
Supplement: Supplementary file 1 — Supporting Information [file 41396_2023_1510_MOESM1_ESM.pdf]

Supporting Information for

Plastoquinone synthesis inhibition by tetrabromo biphenyldiol as a widespread algicidal mechanism of marine bacteria

Zenghu Zhang, Dehai Li, Ruize Xie, Ruoyu Guo, Shailesh Nair, Huan Han, Guojian Zhang, Qun Zhao, Lihua Zhang, Nianzhi Jiao, Yongyu Zhang*

***Correspondence**:
Yongyu Zhang, zhangyy@qibebt.ac.cn

This PDF file includes:

Supporting Materials
Supporting Results
Figures S1 to S11
Legends for Tables S1 to S6

Other supporting materials for this manuscript include the following:
Tables S1 to S6

Supplementary Materials

Isolation and identification of algicidal strains

Synechococcus sp. PCC7002 (obtained from the Pasteur Culture Collection) was cultured in A^+ medium at 26°C with a 12:12 h light:dark cycle of 50 μmol photons m^{-2} s^{-1}, used to isolate algicidal bacteria in this study. Sediment samples, often considered an important source of algicidal bacteria, were collected from the coast of Qingdao (N 36°09′, E120°21′). The bacterial suspensions were obtained by soaking and shaking the sediments in sterile seawater for 30 min and adding them to the exponentially growing *Synechococcus* sp. PCC7002 for one-week cocultivation. After observing algal lysis, the representative bacterial strains were isolated by serial dilution. The algicidal activity was verified by inoculating the bacteria on a *Synechococcus* plate and observing whether there was an algae-lysing zone.

The morphological characteristics of the algicidal strains were observed with an Olympus BX-51 optical microscope and a Hitachi H-7650 transmission electron microscope. Their classifications were confirmed by amplifying the 16S rRNA gene with primers 27F and 1492R. Here, an algicidal bacterial strain *Microbulbifer* sp. RZ01 (belonging to the class *γ-proteobacterium*) was isolated. In addition, another bacterial strain, TB12003, with the same 16S rRNA gene as RZ01 but with a different colony color, was isolated.

Determination of the growth curve of algicidal bacterial strain Microbulbifer sp. RZ01

Colonies grown on solid 2216E, were transferred to 5 mL liquid 2216E and activated at 28 ℃ and 150 rpm for 24 h. Subsequently, the culture was inoculated into 150 mL liquid 2216E at a concentration of 1.0% (v/v), and incubated at 28 ℃ and 150 rpm. The samples were collected every 4 h, and the OD at 600 nm was measured with a spectrophotometer.

Determining how strain RZ01 inhibits the algae and the properties of the algicidal substance

Strain RZ01 was grown to exponential, stationary and decline phases in 20-h, 40-h and 60-h incubations, respectively, after which it was inoculated at a concentration of 5% (v/v) into exponentially growing *Synechococcus* sp. PCC7002. Moreover, in order to To determine the anti-algal mode of the bacteria (i.e., whether the bacteria killed the algae directly or indirectly by secreting substances), the cell-free supernatant and cells of the RZ01 culture were added to the algal cultures to assess their algicidal activity. In brief, strain RZ01 was inoculated into 30 ml of 2216E broth and grown on a shaker at 150 rpm 28 ℃ for 60 h. The cell-free supernatant was collected by centrifugation at 8,000 rpm for 10 min and then passed twice through a 0.22-μm filter (Millipore). The cell pellets were washed twice with sterile seawater and re-suspended in A^+ medium. These different fractions were then added into exponentially growing cultures of *Synechococcus* sp. PCC7002. The algicidal rate was determined as follows: *Algicidal rate* = (FC-FT)/FC x 100%, where FT and FC represent the fluorescence intensity of the algal culture with and without bacteria, respectively. All experiments were performed in triplicate.

To test the thermal stability of the algicidal substance, the cell-free supernatant was incubated at 100 ℃ for 30 min, and then cooled to 28 ℃ before it was added to *Synechococcus* cultures at a concentration of 10% (v/v). To determine the effect of pH on the algicidal substance, the cell-free supernatant was adjusted to pH 1, 3, 5, 7, 9, 11 or 13 using 1 M HCl and 1 M NaOH and incubated for 2 h. It was then adjusted back to the initial pH (pH 8), after which it was added to the *Synechococcus* culture at a concentration of 10% (v/v). Finally, to characterize the molecular size of the algicidal

substance, the cell-free supernatant was sequentially passed through 30 KDa, 10 KDa and 3 KDa pore-size ultrafiltration tubes (Millipore) and centrifuged at 4,000 rpm for 30 min, after which the corresponding filtrates were added to the algal cultures.

Heterologous expression and enzymatic activity assay of homogentisate solanesyltransferase

The *P. tricornutum* homogentisate solanesyltransferase (HST) ORF (lacking a transit peptide sequence) was cloned into the pGEX-6P-1 vector for the expression of HST as a recombinant protein with an N-terminal glutathione S-transferase (GST)-tag, according to the manufacturer's instructions. Protein expression was carried out in *E. coli* Rosetta (DE3); these cells were grown at 37 ℃ in LB medium supplemented with 25 μg/mL chloramphenicol and 100 μg/mL ampicillin. Expression was induced by the addition of IPTG at final concentration of 0.6 mM and the cells were cultured for 16 h at 20 ℃. Subsequently, the cells were collected by centrifugation and lysed with buffer comprising 8 M urea, 50 mM Tris, 300 mM NaCl, and 0.1% Triton X-100, pH 8.0 followed by sonication. The supernatant collected after the centrifugation step contained crude proteins, which were purified with an Ni-NTA column, separated by SDS-PAGE and identified by Western blot. Meanwhile, the *E. coli* cell membranes, (expressing *hst*), were collected from the IPTG-induced *E. coli* cultures by sequential centrifugation at 500 g and ultracentrifugation at 150,000 g.

E. coli-expressed HST activity was determined by testing whether homogentisate and farnesyl pyrophosphate (or geranylgeranyl pyrophosphate) could be catalyzed by HST to form 2-methyl-6-farnesyl-1,4-benzoquinol (or 2-methyl-6-geranylgeranyl-1,4-benzoquinol) (Sadre et al. 2010, DOI: 10.1074/jbc.M110.117929). *E. coli*-expressed HST was assayed for 30 min at 28 ℃ in a 100 μL reaction with purified HST protein, 20 mM $MgCl_2$, 100 μM homogentisate (Macklin), either 200 μM farnesyl pyrophosphate (Sigma-Aldrich) or 200 μM geranylgeranyl pyrophosphate (Sigma-Aldrich) in 50 mM Tris-HCl buffer, pH 7.5. Considering that HST protein might lose its enzymatic activity after purification, *E. coli* membranes containing HST protein were added to the reaction system instead of the purified protein. These enzyme assays were stopped with 100 μL methanol. The reaction was also evaluated by detecting the potential product in the content of the substrate via HPLC according to the method by Shino et al. 2018 (DOI: 10.1584/jpestics.D18-008). The mobile phase was acetonitrile and 0.1% acetic acid at a ratio of 65:35 (v/v). The flow rate was maintained at 1.0 mL/min, and the effluent was monitored using a fluorescence detector with excitation and emission wavelengths of 290 nm and 330 nm, respectively.

Supplementary Results

Comparison of Microbulbifer sp. strains RZ01 and TB12003

The color of colonies of *Microbulbifer* sp. RZ01 grown on solid 2216E was dark yellow, while *Microbulbifer* sp. TB12003 colonies were light yellow (Fig. S1A). In contrast to algicidal bacterial strain RZ01, TB12003 had lost inhibitory activity against algae (Fig. S1B). The inability of the *Microbulbifer* sp. TB12003 to synthesize 4-BP was confirmed by HPLC analysis of the crude extracts of both strains (Fig. S1C).

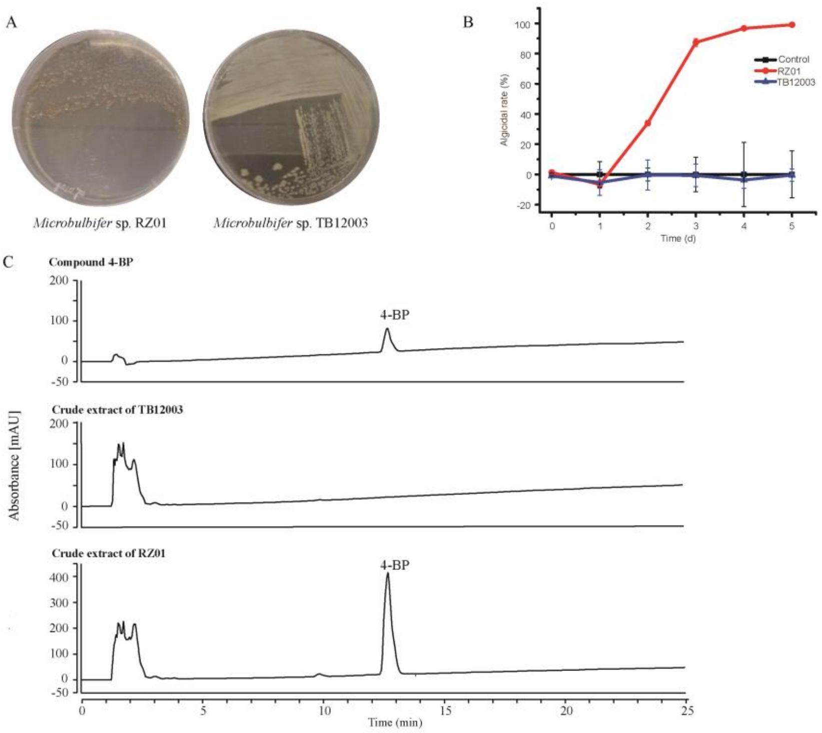

Supplementary Fig. 1 Comparison of *Microbulbifer* sp. strains RZ01 and TB12003 in terms of colony color, algicidal activity and 4-BP production. A: Two strains grown on solid 2216E. B: The algicidal effect of the two strains on *Synechococcus* sp. PCC7002. C: The detection of 4-BP was conducted in both strains using a modified method (Agarwal et al., 2014, DOI: 10.1038/nCHeMBIO.1564). Briefly, 50 mL of bacterial cultures were grown in liquid 2216E at 28 ℃ for 72 h with shaking at 150 rpm. The cultures were extracted with 100 mL of ethyl acetate. The organic layer was dried with flowing nitrogen at 50 ℃. The residue was dissolved in 1 mL of MeOH. The 0.2-μm filtered extracts were injected into an Agilent poroshell 120 EC-C_{18} column by a Thermo Scientific Vanquish HPLC System with a UV detector (254 nm). The solvent A was 0.1% formic acid in water and B was 0.1% formic acid in MeCN. The elution process consisted of a linear gradient from 60% to 100% B over a period of 30 min. The chemically synthesized compound 4-BP was used as a reference standard.

Carbon fixation and carbohydrate pathways of P. tricornutum are affected by 4-BP

KEGG pathway analysis revealed that 4-BP significantly affected Calvin cycle, TCA cycle, glycolysis/gluconeogenesis, and pentose phosphate pathways (Figs. S2-5; Tables S4). Although low concentrations (≤ 1.0 μM) of 4-BP did not kill the cells, the carbon metabolic processes were still significantly affected.

The transcriptional expression of Calvin cycle related genes exhibited differential patterns of expression with the different concentrations of 4-BP (Fig. S2). For example, they were up-regulated by 1.0 μM 4-BP but down-regulated by 3.6 μM 4-BP, suggesting that the effect on the Calvin cycle was dose-dependent. In general, the RuBisCO large subunit gene (*rbcL*) is known to be sensitive to changes in environmental stress, but we did not observe any significant transcriptional changes in *rbcL* due to 4-BP in this study. However, the genes involved in the reduction and regeneration of the Calvin cycle were largely affected (Table S4). For example, the gene that encodes citrate synthase (GeneID: 7195620), which is the key regulatory enzyme of the TCA cycle, was up-regulated (Fig. S4). The pyruvate dehydrogenase complex, which involved in the process from pyruvate to acetyl-CoA,

components coding genes including dihydrolipoic transacetylase (*DHLTA*), dihydrolipoyl dehydrogenase (*DLDH*), and pyruvate dehydrogenase (*PDH*) were significantly up-regulated at 24 h (Table S4). Furthermore, the gene encoding glucose-6-phosphate 1-dehydrogenase (*G6PDH*) (GeneID: 7195268) was significantly down-regulated, indicating that the pentose phosphate pathway (PPP) might be inhibited by a high concentration of 4-BP, especially at 24 h (Fig. S5). Differentially expressed genes of important glycolysis enzymes, such as pyruvate kinase and 6-phosphofructokinase genes, were also detected. The pyruvate kinase coding genes (Fig. S3, Table S4) were up-regulated in most of the 4-BP treated samples except when incubated with 3.6 μM for 72 h. These results indicate that 4-BP disrupted carbohydrate metabolism.

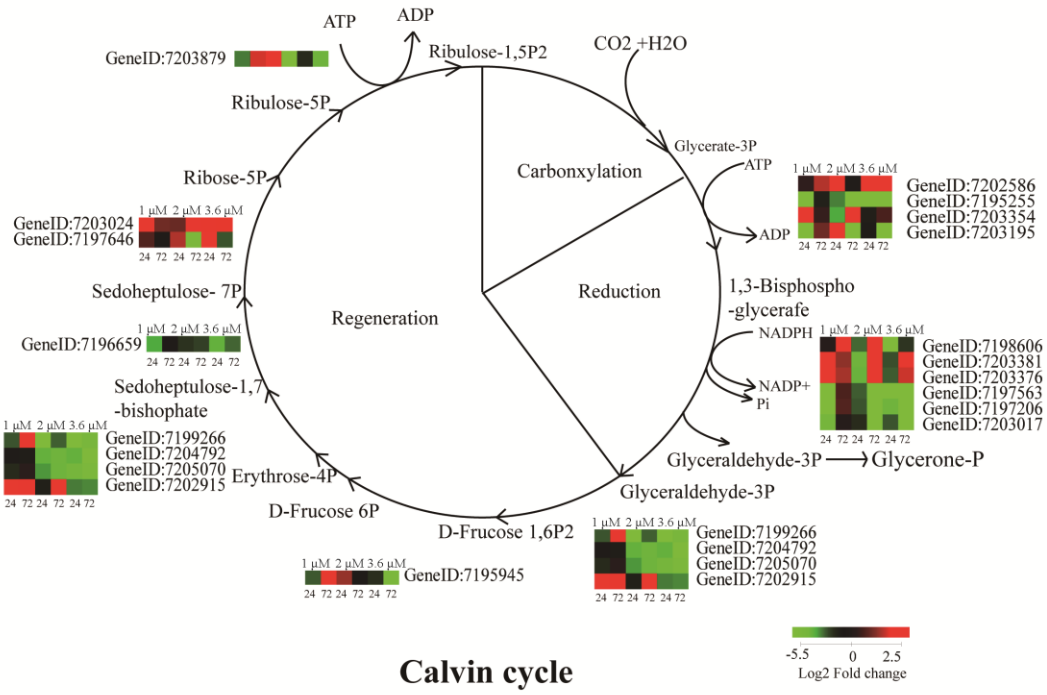

Supplementary Fig. 2 Regulation of the Calvin cycle at the transcriptional level by 4-BP in *Phaeodactylum tricornutum* CCMP2561.

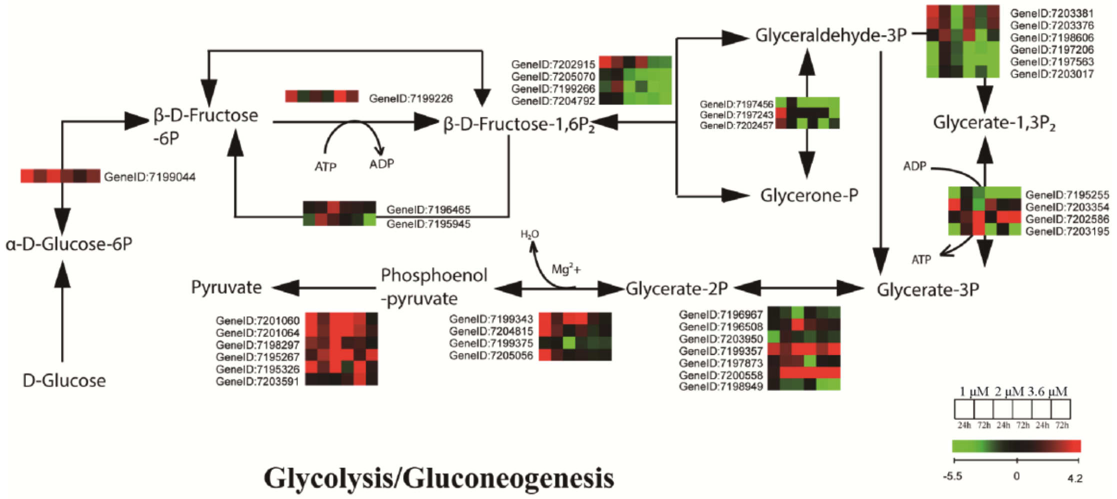

Supplementary Fig. 3 Regulation of glycolysis and gluconeogenesis at the transcription level by 4-BP

in *Phaeodactylum tricornutum* CCMP2561.

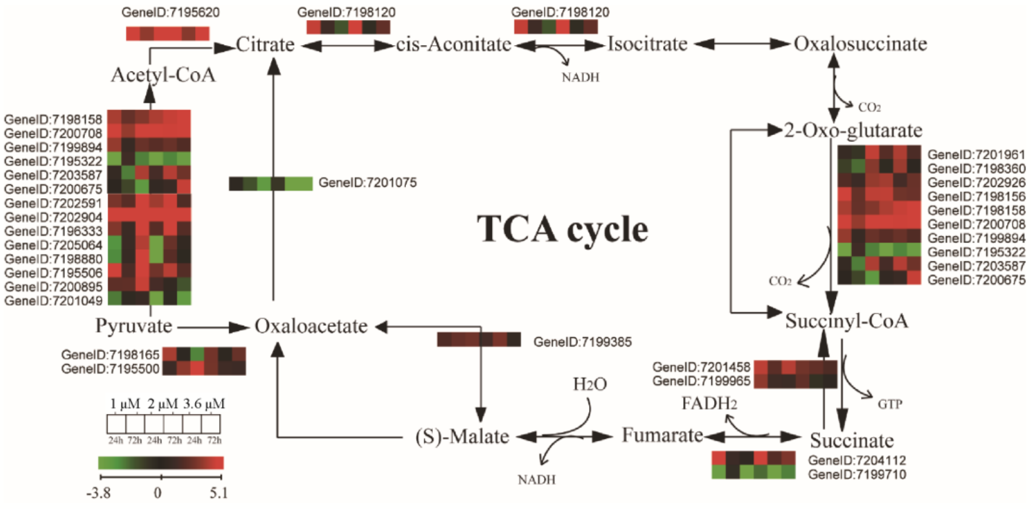

Supplementary Fig. 4 Regulation of the TCA cycle at the transcription level by 4-BP in *Phaeodactylum tricornutum* CCMP2561.

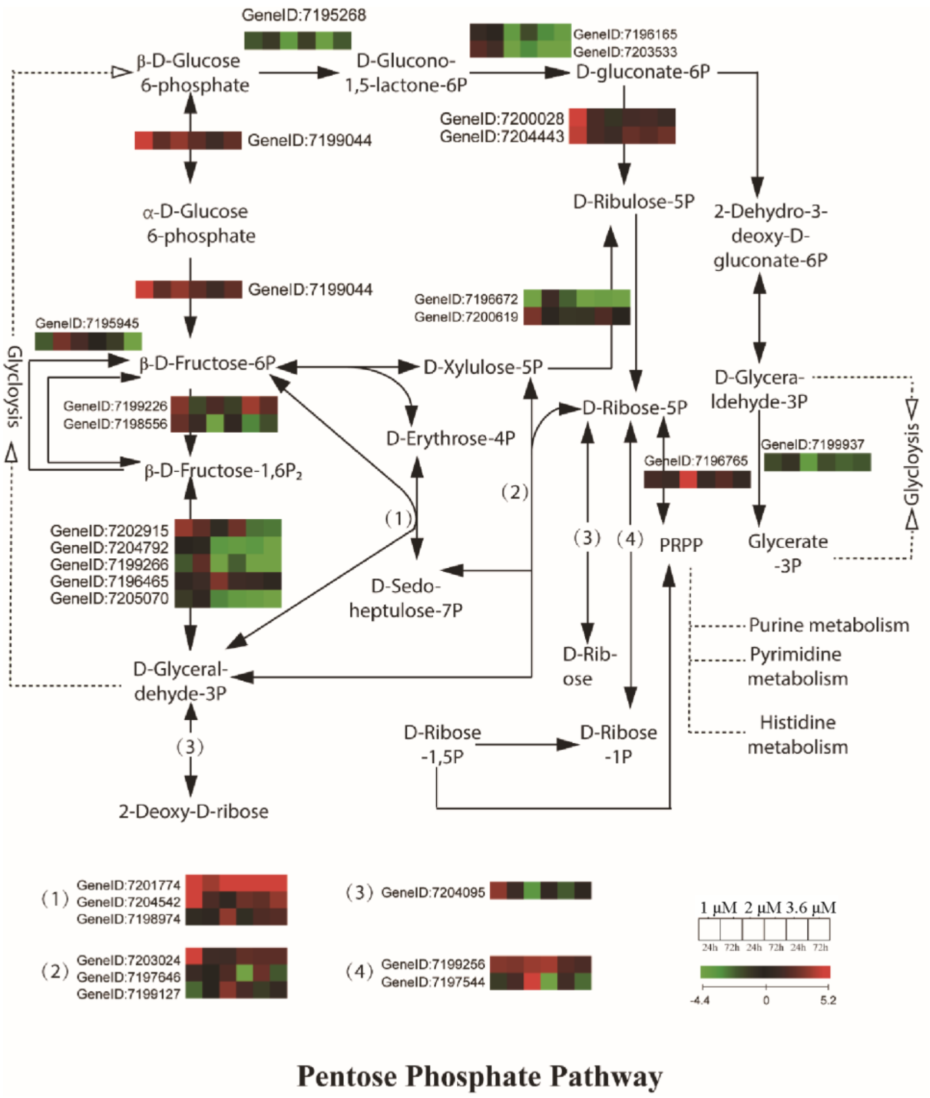

Supplementary Fig. 5 Regulation of the pentose phosphate pathway at the transcription level by 4-BP in *Phaeodactylum tricornutum* CCMP2561.

4-BP increases oxidative stress in P. tricornutum

A significant increase in ROS was detected at 4-BP concentrations of 2 μM, 3.6 μM or 6 μM, but not at lower concentrations (i.e., 0.5 μM and 1.0 μM) (Fig. S6). At 3.6 μM or 6 μM 4-BP, the ROS level gradually increased f between 4 h and 24 h, but then it decreased. At 2 μM 4-BP, the highest concentration of ROS was detected 8 h after the start of treatment and then it decreased. At the transcriptomic level, genes encoding glutathione transferase (GeneID:7196284; GeneID:7202565), and ascorbate peroxidase (GeneID:7201607) were increased, and many of genes involved in cell redox homeostasis also responded to 4-BP (Table S4). These results suggest that 4-BP might disturb the redox homeostasis in *P. tricornutum*, and in turn *P. tricornutum* activates antioxidants to mitigate the 4-BP-induced oxidative stress.

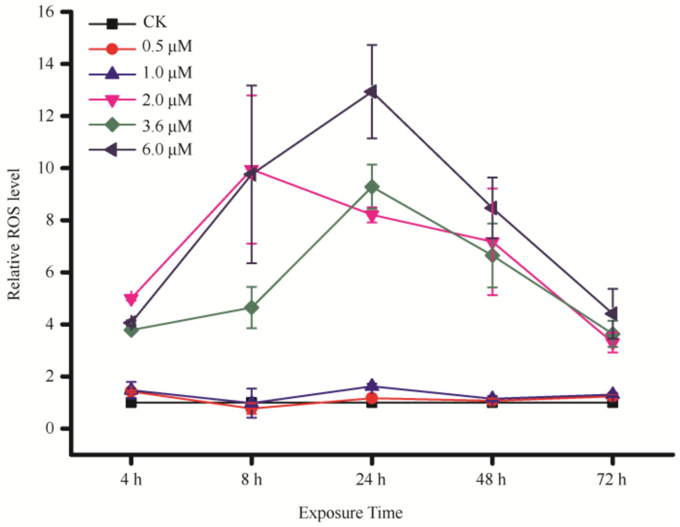

Supplementary Fig. 6 Effect of 4-BP on the amount of ROS in *Phaeodactylum tricornutum* CCMP2561.

Attempts to validate HST enzyme activity in vitro

We obtained the HST protein by heterologous expression (Fig. S7A-C), but when performing *in vitro* enzyme activity assays, neither the purified protein nor the collected HST-containing *E. coli* membrane protein could catalyze the reaction between homogentisic acid and either farnesyl diphosphate or geranylgeranyl pyrophosphate, and there was no reduction in the content of homogentisic acid or increase in the formation of potential products (Fig. S7D-E). Although some studies have validated HST protein activity in *Arabidopsis thaliana*, we speculate that there may be additional dependencies on the activity of HST in diatoms that require further in-depth investigation.

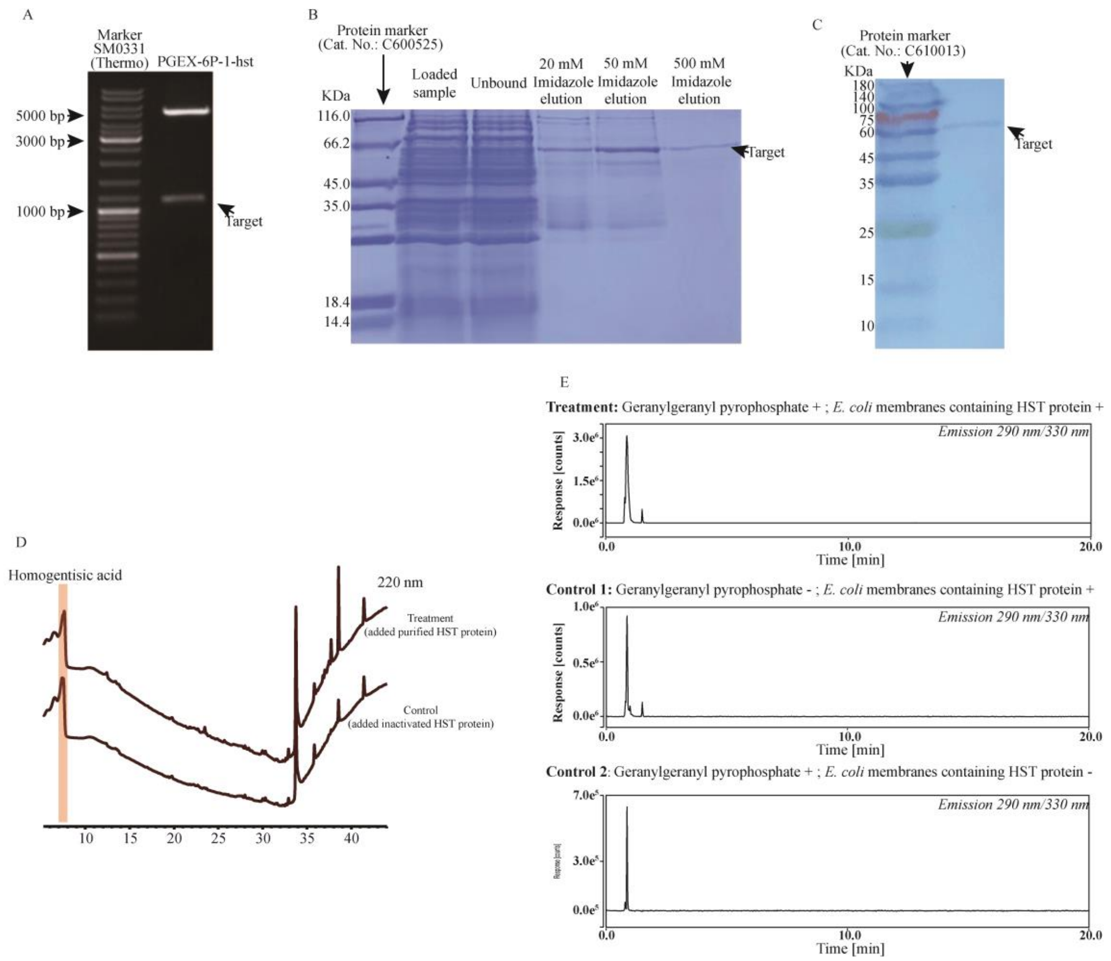

Supplementary Fig. 7 Heterologous expression and enzyme activity assay of homogentisate solanesyltransferase. A: Agarose gel electrophoresis of the recombinant plasmid digested with enzymes. B: SDS-PAGE analysis of the fusion protein purified by nickel agarose affinity chromatography. C: Western Blot analysis of the final purified protein. D: HPLC detection of homogentisic acid in the reaction mixture with and without active HST protein. The 100 μL reaction mixture contained homogentisic acid (100 μM), farnesyl pyrophosphate (200 μM), $MgCl_2$ (20 mM), Tris-HCl Buffer (pH 7.5) and purified protein. The reaction was carried out at 28 ℃ for 2 h and then stopped by adding an equal volume of methanol. The reaction mixture was centrifuged, and the effluent was measured with a UV detector at 220 nm. In the control group, the purified protein was first treated with boiling water and then cooled before being added to the reaction mixture. E: The potential products of the HST-mediated reaction system were detected by HPLC. The 200 μL reaction mixture of the treatment group contained homogentisic acid (200 μM), geranylgeranyl pyrophosphate (200 μM), $MgCl_2$ (20 mM), Tricine-NaOH buffer (50 mM, pH 8.5) and 1.5 mg/mL membrane proteins. The reaction was carried out at 28 ℃ for 30 min and then stopped by adding 200 μL of 20 mM ascorbic acid and 80 μL of 0.1 M acetic acid. The reaction system was centrifuged, and the effluent was detected using a fluorescence detector with excitation and emission wavelengths of 290 nm and 330 nm, respectively. Controls 1 and 2 did not contain geranylgeranyl pyrophosphate and membrane proteins, respectively.

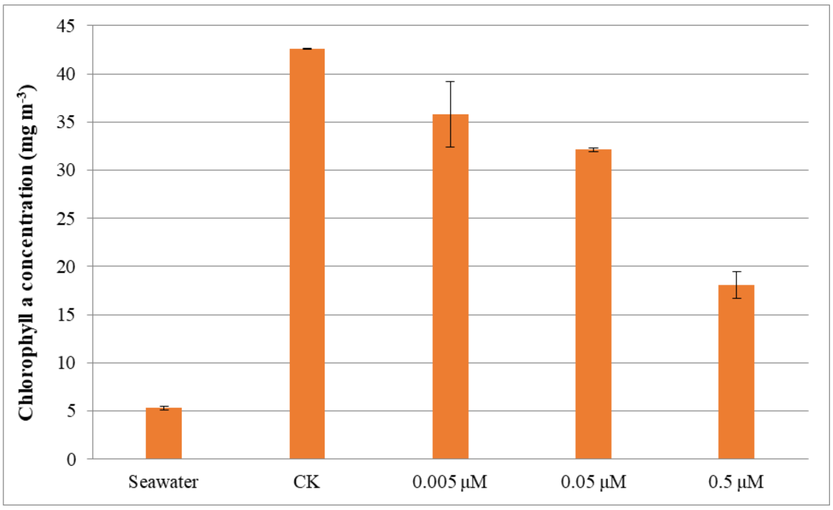

Supplementary Fig. 8 Effect of 4-BP on chlorophyll *a* concentration in a simulated algal blooming seawater. "CK" represents simulated algal bloom seawater without 4-BP addition; "0.005, 0.05, 0.5 µM" represents simulated algal bloom seawater treated with 0.005, 0.05, 0.5 µM concentrations of 4-BP for three days, respectively.

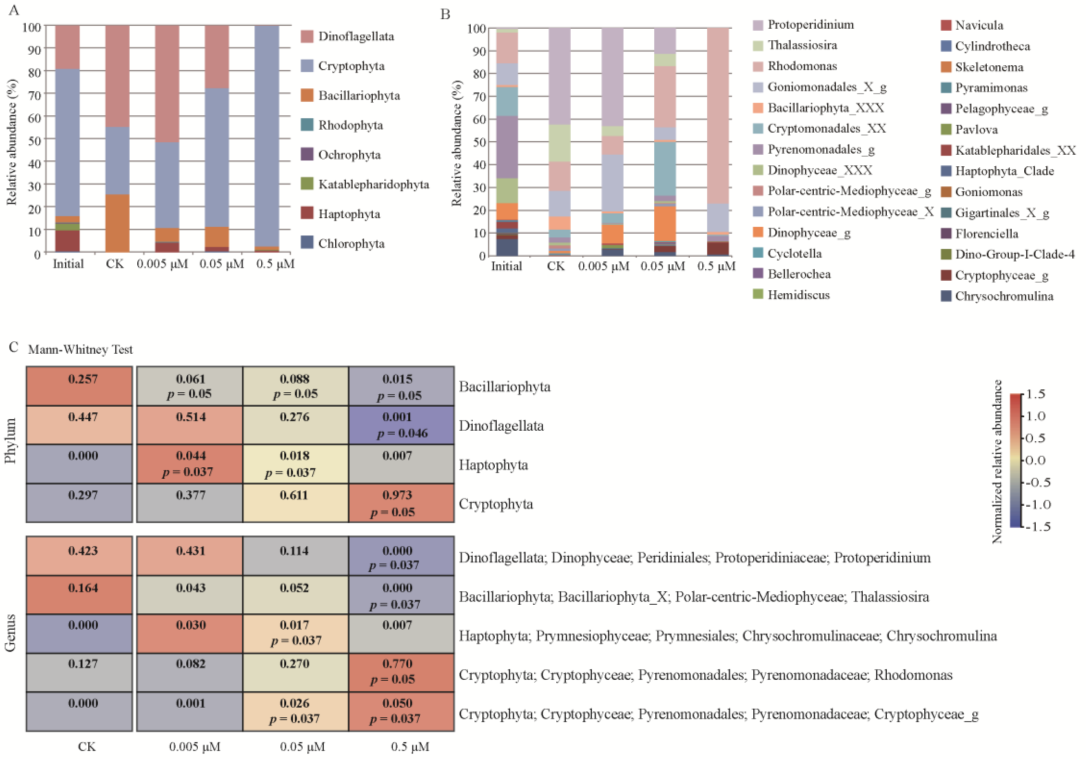

Supplementary Fig. 9 Comparison of the phytoplankton community structure in the control and 4-BP-treated groups at the phylum (A) and genus (B-C) level. (A and B) "Initial" represents the phytoplankton community structure at the beginning of the experiment. "CK, 0.005, 0.05, and 0.5 µM"

represent the phytoplankton communities after three-day treatment with 0.005, 0.05, and 0.5 μM 4-BP, respectively. Triplicates of each sample were sequenced separately and each column showed the mean relative abundance of the taxa. (C) Statistical analysis between control (CK) and treatment groups (0.005, 0.05, and 0.5 μM) using Mann-Whitney test. The number in each square showed the mean relative abundance of the taxa. Treatments with significant differences are labeled with their *p*-values.

Growth characteristics of Microbulbifer sp. RZ01

Microbulbifer sp. RZ01 was cultured on solid 2216E at 28 ℃ for 4 days and formed colonies with a diameter of 2 to 3 mm that were oval in shape with regular edges, light brown in colour, and smooth and moist in appearance (Fig. S10a). The cells of the strain were rod-shaped (0.3-0.6 μm wide and 3-7 μm long), without flagella (Fig. S10b). After strain *Microbulbifer* sp. RZ01 was transferred to fresh liquid 2216E, its growth lag period lasted to 12 h, and the exponential growth period was from 12 h to 32 h. After which, the cell density remained stable until 50 h, and then the strain entered the decay phase (Fig. S11).

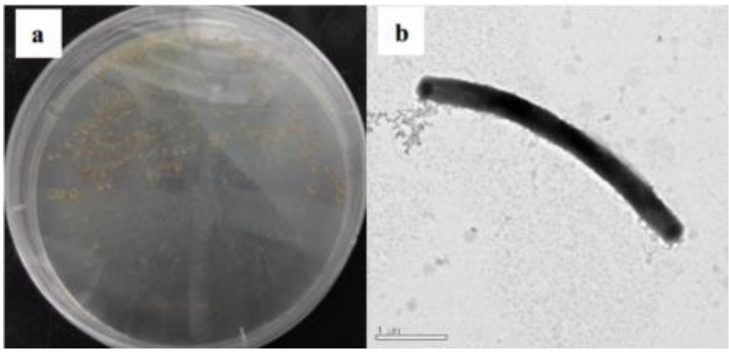

Supplementary Fig. 10 Colony (a) and cell (b) morphology of strain *Microbulbifer* sp. RZ01. The size of scale bar in panel b is 1 μm.

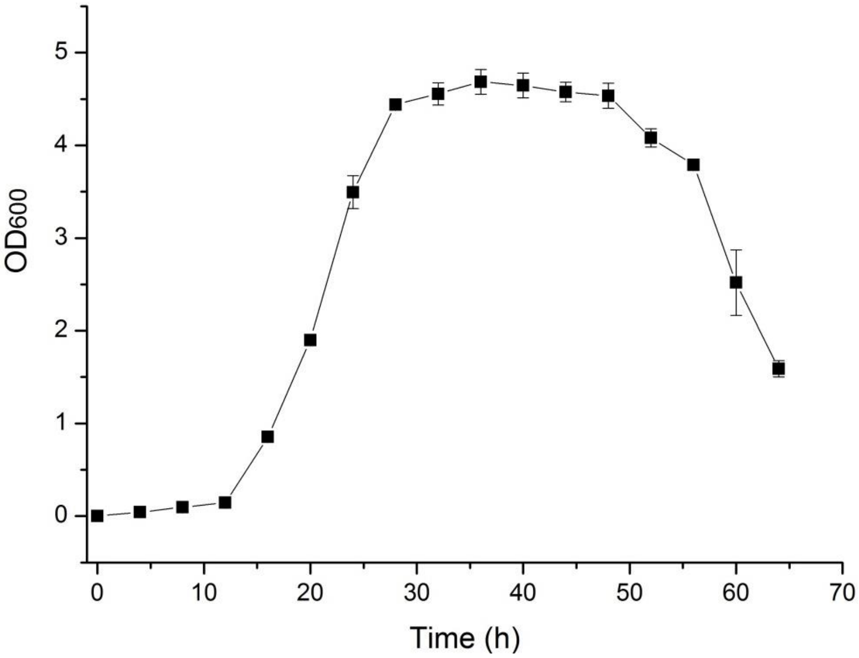

Supplementary Fig. 11 The growth curve of strain *Microbulbifer* sp. RZ01

Nucleic acid sequence of the homogentisate solanesyltransferase gene from *Phaeodactylum tricornutum* CCMP2561 and the corresponding amino acid sequence

> homogentisate solanesyltransferase gene

ATGGGCTCCATAACGTGGTCAGTCCTCGTCGTTATGTTGCTGTCGGCGACATCGAGTGTATC
GGCATTCTCGCATGGCCAACCACAAAGAAGCTCTTTCCCATCCGCATGCTTGTTTCAGCTTC
GACTCCCAAAAGCTACTGGGCCCCAATTACCATTGTATATGATGCCTGTGGCCCGTTCAATG
TCGGTGGAGGGCGACTTCGATCCCGACGCCGTCGCTCGGGCTCGTGAAGAAGCGCAGTTG
GGATCGCTCAAAGCCAAACTCCGGGCCCTCTACAAGTTCACGCGTCCCCACACAATTCGCG
GCACCATCTTAGCTTCAATTGCCGGGACGACTCGTGCTCTGATAGATACGCCGGGCGCGATT
GCTAATGCCAACTGGAGTATCATGCTGCCACGAGCCCTGATCGGCATGACTGCCTTATTGCT
CGGCAACGCCTTTATTGTCGGCATCAATCAGATTTACGACGAATCCATTGACAAGCTCAATA
AGCCGTTCTTGCCGGTGGCTAGCGGAGAAATGTCGAAGCGATTTGCTTGGGTCGCGGTTGT
CGTGTCCGGCTTGGTTGGACCGTCGTTGGTTTATCAATTCTTTCCGCGTCTTCTCTTCAAGTT
ATATAGYATGGGAATTGTTCTCGGCGGCATCTACTCTGTGCCTCCCATTCGGACGAAGAAGA
ATCCCGTGCTGGCTGGGCTCACAATTGCYACCGTCCGAGGCTTTCTGCTCAATTTTGGCGTC
TACTATGCCGTCAAGGACGCCATCAACGCGCCATTTGTWTGGTCACCTAAGGTGGCCTTTAT
TGCACGGTTCATGACGGCTTTCGCGACCGTGATTGCCGTCACCAAGGATCTTCCCGATATTG
AAGGAGACAAGGCTTTCCAGATAGATACTTTTGCTACCAAGGTAGGTGTCGCGCGCATCGC
GAAAGGTGCATCCGTCTGTCTGCTGCTAAATTATGTGCACGCCGTTGCAACTGGTGTGCGA
GCGGCCACCGGAACGTTCAACCTCGTCCCGATGATTGGAGGGCACGTCGCCCTCGCCTCTA
TGCTGGCGTATCGCTTCCGACAACTAGATCCCGACTCGATGCCATCCGTCAAACTCTTTTATA
AGCACGTGTGGGATTTGTTTTATCTTGAATATGGGCTCTACACTTTAATTTAA

> homogentisate solanesyltransferase protein

MGSITWSVLVVMLLSATSSVSAFSHGQPQRSSFPSACLFQLRLPKATGPQLPLYMMPVARSMSV
EGDFDPDAVARAREEAQLGSLKAKLRALYKFTRPHTIRGTILASIAGTTRALIDTPGAIANANWS
IMLPRALIGMTALLLGNAFIVGINQIYDESIDKLNKPFLPVASGEMSKRFAWVAVVVSGLVGPSL
VYQFFPRLLFKLYXMGIVLGGIYSVPPIRTKKNPVLAGLTIATVRGFLLNFGVYYAVKDAINAPF
VWSPKVAFIARFMTAFATVIAVTKDLPDIEGDKAFQIDTFATKVGVARIAKGASVCLLLNYVHAV
ATGVRAATGTFNLVPMIGGHVALASMLAYRFRQLDPDSMPSVKLFYKHVWDLFYLEYGLYTLI
*

Supplementary table 1 Algal strains used to assess the anti-algae spectrum of *Microbulbifer* sp. RZ01

Supplementary table 2 The 13 metagenomic assembled genomes containing all three genes of 4-BP were widely distributed at 35 sampling sites. Note: The metagenomic assembled genome (MAGs) data and sampling site information cited from Tully et al. 2018, DOI: 10.1038/sdata.2017.203.

Supplementary table 3 The 2750 whole genomes involving 26 bacterial taxa were used to screen for potential 4-BP synthesizing strains

Supplementary table 4 Summary of differentially expressed genes in *Phaeodactylum tricornutum* under 4-BP treatments.

Supplementary table 5 Overview of HST protein-molecule interactions based on Protein-Ligand Interaction Profiler (PLIP) analysis

Supplementary table 6 Primers, PCR reaction system and PCR reaction procedure for amplifying algicidal related genes bp608-610.
